# Effectiveness of a smartwatch-based feedback system in improving cardiopulmonary resuscitation quality: a simulation study

**DOI:** 10.1016/j.resplu.2025.101114

**Published:** 2025-09-30

**Authors:** Leandro Menezes Alves da Costa, Rafael Otto Schneidewind, Rogerio Ferrari Peron, Thiago Timmerman, Fabio de Cerqueira Lario, Frederico Rafael Moreira, Rafael Amorim Belo Nunes, Thiago Luis Scudeler

**Affiliations:** Hospital Alemão Oswaldo Cruz, São Paulo, Brazil

**Keywords:** Cardiopulmonary resuscitation, CPR Feedback, Basic Life Support, Medical education, Training

## Abstract

Background High-quality cardiopulmonary resuscitation (CPR) is a critical determinant of survival in cardiac arrest. Although current guidelines endorse the use of feedback devices to improve CPR performance, their limited availability in clinical settings poses a significant barrier. To address this gap, we developed a CPR feedback application for smartwatches, leveraging wearable technology to provide real-time, user-friendly guidance for chest compressions. Our study was to assess whether a commercially available smartwatch could function effectively as a real-time CPR feedback device and improve compression quality.

Methods In this single-center, sequential trial, 90 healthcare professionals performed two 2-minute sessions of chest compressions on a manikin, separated by a 2-minute rest. The first session was performed without feedback, and the second using the VIMO feedback application integrated into an Apple Watch Series 9. The primary outcome was the Overall Performance Score, a composite of rate and depth effectiveness.

Results Use of the smartwatch-based feedback system significantly improved CPR quality. The Overall Performance Score increased from a median of 82.5 % (IQR 49.0–97.0) without feedback to 97.0 % (IQR 80.0–99.0) with the device (*p* < 0.001). Mean depth effectiveness (82.0 % vs. 71.0 %) and median rate effectiveness (94.5 % vs. 62.5 %) also improved significantly (both *p* < 0.001).

Conclusion The use of a feedback-enabled smartwatch improved CPR quality metrics, including compression depth and rate, among healthcare professionals. Nevertheless, these findings should be interpreted with caution when extrapolating to real-world cardiac arrest scenarios. Further research is warranted to assess the effectiveness of this approach during actual resuscitations and among lay rescuer populations.

## Introduction

High-quality cardiopulmonary resuscitation (CPR) and early defibrillation are the cornerstones of survival in cardiac arrest.[1], [2] Despite improvements in resuscitation science and emergency care systems, survival rates after out-of-hospital cardiac arrest (OHCA) remain low in most regions, with global estimates ranging from 8 % to 10 %,[3], [4], [5] and only a few countries reporting rates as high as 16 % to 20 %.[6], [7]

Notable enhancements, including widespread training programs to improve CPR performance, early recognition of cardiac arrest, and dissemination of automated external defibrillators (AEDs) in high-density public settings, have significantly contributed to reducing morbidity and mortality. The implementation of public access AEDs has been associated with improved survival rates in OHCA, particularly when used by bystanders before emergency medical services arrival.[8], [9] While the widespread deployment of public access AEDs has contributed to improved survival following OHCA, optimizing the quality of chest compressions remains another critical link in the chain of survival with significant potential to further enhance patient outcomes.^10^ High-quality CPR, characterized by adequate compression depth, correct rate, full chest recoil, and minimal interruptions, has been consistently associated with improved survival and neurological outcomes following cardiac arrest.[11], [12], [13], [14], [15] CPR feedback devices are increasingly recognized as valuable adjuncts to support the delivery of high-quality chest compressions, although current resuscitation guidelines do not yet strongly recommend their routine use across all settings. Automated real-time feedback systems are particularly valuable, as they improve both the acquisition and retention of CPR skills, enhancing the overall quality of resuscitation efforts.^16^

Real-time feedback devices have been shown to improve CPR performance by providing immediate correction of compression depth, rate, and other critical quality parameters, thereby enhancing adherence to guideline-recommended targets during training and simulation scenarios.[11], [12], [17], [18] These tools, ranging from simple metronomes to sophisticated audiovisual systems, have consistently demonstrated improved CPR quality during both training and clinical scenarios. Feedback is given via visual or auditory cues, enabling rescuers to adjust their technique according to resuscitation guidelines. Comparative research has assessed the effectiveness of various feedback modalities, including metronomes, audiovisual systems, smartphone applications, portable devices, and AEDs equipped with integrated CPR feedback functionalities.[19], [20], [21]

Despite their demonstrated potential to improve PCR quality metrics in simulation and certain clinical settings, the widespread adoption of these devices has been hindered by several limitations, including high costs, single-use constraints, cumbersome designs, and reliance on integration with defibrillation paddles.[22], [23]

Smartwatches equipped with accelerometers and gyroscopes offer a novel, widely accessible platform for CPR feedback. These wearable devices are already popular among the general population and can provide real-time biomechanical monitoring. Previous simulation-based studies have shown promising results using smartwatches to improve CPR quality among laypeople and healthcare providers.[24], [25], [26] However, most studies have been limited to small sample sizes, older smartwatch models, and lack standardization in outcome measurement.

Unlike previous studies that evaluated earlier-generation smartwatch-based feedback systems, our study is the first to assess CPR quality using the Apple Watch Series 9, featuring advanced motion sensors with higher sampling rates and improved measurement accuracy. In addition, we utilized a newly developed, internally validated feedback app (VIMO CPR feedback App), designed to leverage these hardware improvements to provide real-time, dual-mode (audio and visual) feedback to rescuers.

To our knowledge, no study has comprehensively evaluated a smartwatch-based CPR feedback system using the latest generation devices in a representative healthcare provider sample. Our study aims to evaluate the effectiveness of a feedback-enabled smartwatch in improving CPR quality among healthcare professionals during simulated resuscitation.

## Methods

We conducted a single-center, sequential simulation study involving 90 participants between August and October 2024. All participants were healthcare professionals from Hospital Alemão Oswaldo Cruz (São Paulo, Brazil), including physicians, nurses, nurse technicians, physiotherapists, and medical students.

Each participant performed two consecutive 2-minute sessions of chest compressions on a manikin, separated by a 2-minute rest period. The first session was performed without feedback, and the second utilized the VIMO CPR feedback application (VIMO SA, São Paulo, Brazil) integrated into an Apple Watch Series 9 (Apple Inc., Cupertino, CA, USA).

Given the paired study design, each participant served as their own control, inherently minimizing confounding by fixed individual characteristics (e.g., age, sex, baseline experience). To explore potential effect modification, we conducted subgroup analyses stratified by professional groups (doctors and other health care professionals) and prior CPR training level (None, Basic, Advanced). However, the study was not powered for formal interaction testing. This stratification was intended solely to explore potential effect modification by baseline skill level and does not adjust for potential order bias. Given the non-randomized, fixed-order design, the study cannot definitively account for time-related confounding such as learning or practice effects between the two sequential CPR trials. CPR quality was assessed using a QCPR-enabled manikin (Little Anne QCPR, Laerdal Medical, Stavanger, Norway) and its associated SkillReporting software. Simulations were conducted individually in a dedicated room, with the manikin placed on a hospital bed to simulate a clinical environment. Participants were evaluated in sequence and remained blinded to the performance of others to minimize potential bias.

### Smartwatch feedback system

The CPR feedback system was based on the VIMO CPR Feedback App, developed for the Apple Watch Series 9. The app processes real-time data from the smartwatch's built-in accelerometer and gyroscope to calculate chest compression rate and depth. It provides both visual (numeric display and color-coded indicators) and auditory feedback (metronome sounds). Prior to the study, the system underwent internal validation using a Little Anne QCPR manikin, demonstrating satisfactory measurement accuracy for both compression depth and rate across varying frequencies (80–140 compressions/min). A detailed description of the development and validation procedures is available in the Supplementary Material.

### Feedback system interaction

The VIMO CPR Feedback App provided real-time feedback using both visual and auditory modalities. Visual feedback included continuous numeric display of compression rate (compressions per minute) and estimated compression depth (centimeters), along with color-coded indicators (green: optimal; yellow: marginal; red: out of target range) based on ERC 2021 Guidelines.^12^

**Fig. 1:**
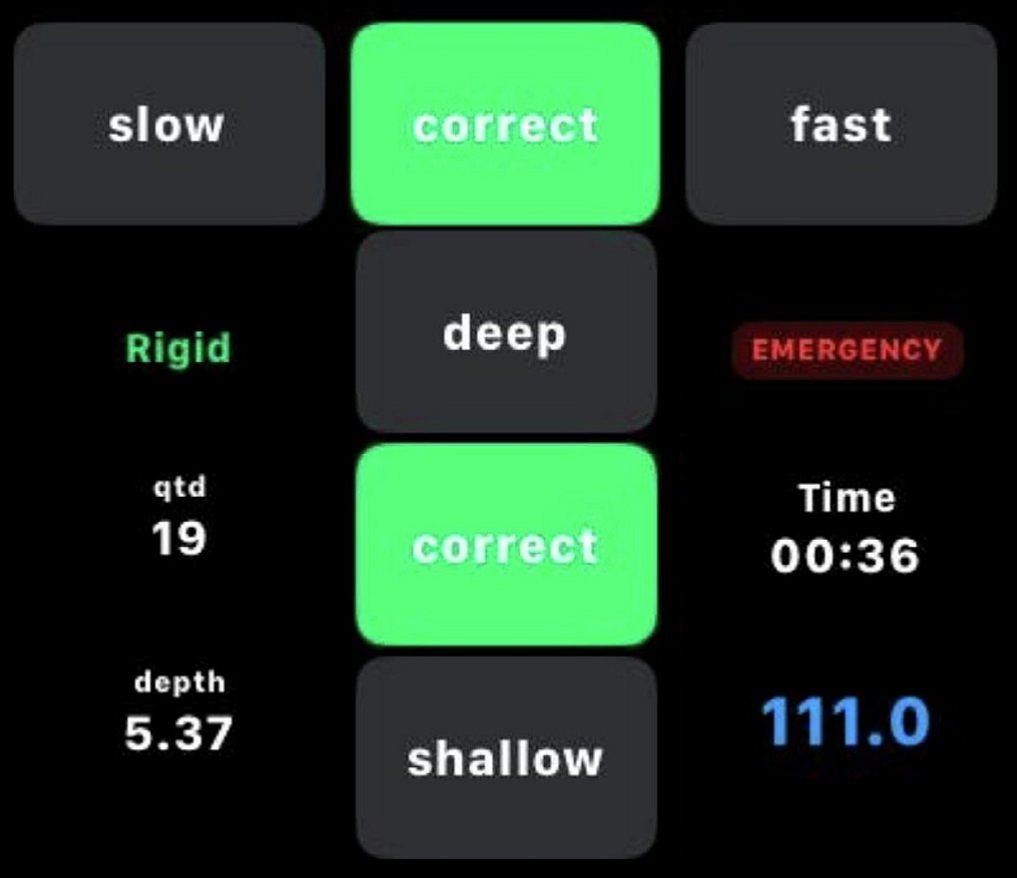
The VIMO CPR System provides real-time visual feedback on compression rate and depth through a smartwatch interface.

The display is organized in a two-axis grid: the horizontal axis represents compression rate, classified as slow (<100 compressions per minute), correct (100–120 compressions per minute), or fast (>120 compressions per minute); and the vertical axis represents compression depth, classified as shallow (<5 cm), correct (5–6 cm), or deep (>6 cm). Additional indicators include the cumulative number of compressions delivered and the total elapsed time of the resuscitation session (Fig. 1).

Auditory feedback was provided through a metronome tone set at 110 beats per minute, emitted by the Apple Watch speaker. Participants were instructed to synchronize their compressions with the metronome and adjust depth according to the visual color indicators. No additional verbal coaching was provided during the scenarios.

### Participant recruitment and eligibility criteria

Participants were recruited between August and October 2024 at Hospital Alemão Oswaldo Cruz (São Paulo, Brazil) using convenience sampling via institutional emails, flyers, and verbal invitations during educational sessions. Inclusion criteria were: (1) age ≥18 years, (2) active status as a healthcare professional (physician, nurse, physiotherapist or medical student), (3) physical ability to perform 2 min of chest compressions, and (4) provision of written informed consent. Exclusion criteria included: (1) musculoskeletal or neurological limitations precluding CPR performance, (2) cardiorespiratory conditions limiting exertion, (3) language barriers or cognitive impairment affecting comprehension of study procedures. Ethnicity was self-reported by participants using predefined categories based on the Brazilian Institute of Geography and Statistics (IBGE) classification system, which is widely used in Brazilian epidemiological research and national census data.

### Primary outcome

The primary outcome was the Overall Performance Score, defined as a measure combining the percentage of effective compression depth range and rate. Secondary outcomes included individual CPR quality metrics: percentage of optimal displacement, percentage of adequate chest recoil, percentage of adequate depth, percentage of correct rate compressions, average compression rate and total number of compressions,

### Overall performance score (OPS)

The OPS was calculated as the arithmetic mean of two independent variables: (1) the percentage of chest compressions performed within the guideline-recommended depth range (5–6 cm) and adequate chest recoil, and (2) the percentage performed within the recommended rate range (100–120 compressions per minute). This composite score provides a single summary percentage (range: 0–100 %) reflecting overall adherence to guideline targets for compression depth and rate. This metric was developed specifically for this study and has not been externally validated.

### CPR quality metrics

All CPR quality parameters were evaluated against internationally recommended target ranges.[11], [12] The target compression rate was defined as 100 to 120 compressions per minute, while the target compression depth was set between 5 and 6 cm of chest depression. Full chest recoil was characterized by the complete release of pressure between compressions, with no residual leaning force applied. Correct hand position was defined as the placement of both hands over the lower half of the sternum during each compression.

The data provided by the Laerdal manikin for CPR quality assessment include: percentage of adequate recoil, percentage of adequate depth, percentage of adequate compression rate, and the average compression rate. As compression quality is related to two factors—adequate recoil and adequate depth—we created a new variable, termed optimal displacement, to represent this relationship more precisely. Furthermore, we combined the variable “average percentage of adequate compression rate” with the optimal displacement variable to generate a summary measure of CPR quality, defined as the Overall Performance Score (OPS). Formula:

OPS=%OptimalDisplacement+%CorrectCompressionRate/2

Optimal Displacement: a composite variable combining the proportion of compressions with adequate depth and the proportion with full chest recoil, reflecting biomechanically correct compressions. Formula:

OptimalDisplacement=%Adequatecompressiondepth+%Compressionrecoilquality/2

Correct hand position: percentage of compressions performed with both hands placed over the lower half of the sternum, as detected by the Laerdal Little Anne QCPR manikin’s algorithm. Notably, although the Laerdal software provides an “overall quality of compressions” metric, we considered it less reliable; therefore, we chose to construct the primary endpoint metric (OPS) using the three main independent parameters. This approach is consistent with previous publications that have employed similar composite measures, described as “overall quality”.[16], [27]

### Pre-intervention knowledge assessment

Prior to the simulation scenarios, participants completed a structured pre-intervention questionnaire designed to evaluate their baseline theoretical knowledge of recommended CPR parameters according to international guidelines. This questionnaire was developed by the research team and reviewed by two CPR education experts for content validity.

The questionnaire consisted of four multiple-choice questions, each focusing on a key component of high-quality CPR: Target compression rate and target compression depth. The need for complete chest recoil and correct hand position was described as perception of CPR metrics. Each participant’s score was calculated as the number of correct responses (ranging from 0 to 4). This Information was used solely for descriptive purposes in the baseline characteristics table and not for inferential analyses.

### Sample size estimation

The sample size was determined a priori based on a paired design, reflecting the within-subject nature of our fixed-order protocol. Given the short rest interval between trials, we anticipated that both fatigue carryover and a potential learning effect could attenuate the magnitude of the observed improvement. We therefore adopted a conservative expected absolute increase in OPS of 5 percentage points, with an assumed standard deviation of within-subject differences of 15 percentage points. Using a two-sided significance level (α) of 0.05 and 80 % statistical power, the sample size formula for the paired-sample *t*-test indicated a minimum requirement of 71 participants. In the absence of directly comparable prior effect size estimates in similar experimental settings, and to account for potential attrition and unanticipated variability, we inflated the target by approximately 25 %, yielding a planned enrollment of 90 participants.

### Post hoc power analysis

The post hoc power analysis conducted showed that the achieved power for the primary outcome (OPS) comparison was 99.9 % for an effect size dz of 0.72 using Wilcoxon signed rank test. G*Power's built-in calculator was applied to determine the effect size dz from differences (mean of difference (value 18.73) and standard deviation of difference (value 25.93). Other input parameters were 2-sided hypothesis test, alpha significance level of 5 %, sample size of 90. We also assumed in this power calculation the Parent distribution parameter (variable distribution) as minARE (minimum absolute relative error). This is a robust option that doesn't assume a specific distribution but uses a minimum absolute relative error approach. All calculations were conducted using G*Power software version 3.1.9.7.[28], [29]

### Statistical analysis

Clinical and sociodemographic variables were reported using median with interquartile range (IQR) for non-normal distributed data, and absolute and relative frequencies for categorical variables.^30^

All outcomes were calculated as the absolute difference between the measurements obtained *with* and *without* the VIMO feedback system. Paired comparisons were performed using either the paired Student’s *t*-test or the Wilcoxon signed-rank test, depending on the normality of the differences.^31^

Normality assumptions were assessed through visual inspection of histograms and the Shapiro–Wilk test.^32^

Results analyzed using the paired *t*-test were reported as mean ± standard deviation (SD), mean difference, and 95 % confidence intervals (95 % CI). Outcomes analyzed with Wilcoxon signed-rank test were reported as median (IQR) by paired group and median difference (95 % CI). The median difference was estimated using the Hodges–Lehmann (HL) method.

Comparisons of quantitative outcomes between two independent groups were conducted with Wilcoxon rank-sum tests, while comparisons across three groups were analyzed using the Kruskal–Wallis test (KW). When KW test was significant, post-hoc pairwise comparisons were performed using the Dwass–Steel–Critchlow–Fligner (DSCF) test.[33], [34], [35] These results were presented as medians with IQRs.

All tests were two-sided, and p-values <0.05 were considered statistically significant. Statistical analyses were performed using SAS version 9.4 (SAS Institute Inc., Cary, NC).

### Subgroup analyses

Exploratory, post-hoc subgroup analyses were conducted to evaluate whether participant characteristics—specifically, professional category (physician, nurse, student) and prior CPR training level (None, Basic, Advanced)—modified the effect of the feedback intervention on CPR quality outcomes. These analyses were not pre-specified in the original study protocol and were not accounted for in the sample size estimation. Therefore, all subgroup findings should be interpreted as exploratory and hypothesis-generating.

Given the exploratory nature of secondary outcomes and subgroup analyses, no formal correction for multiplicity was applied. The primary endpoint (OPS) was the only outcome with pre-specified statistical testing. All other results should be interpreted as exploratory and hypothesis-generating, acknowledging the increased risk of Type I error.

### Ethical approval and informed consent

This study was approved by the Institutional Review Board of Hospital Alemão Oswaldo Cruz (Approval number: 2024-0801). All participants provided written informed consent before participation. The study was conducted in accordance with the Declaration of Helsinki and local regulatory standards.

### Ethical considerations

To mitigate potential conflicts of interest, all CPR quality data were collected via an independent, validated manikin feedback system. Data export and statistical analyses were conducted by investigators with no financial or advisory ties to the device manufacturer. The authors retained full independence in data interpretation and manuscript preparation.

## Results

Baseline demographic and professional characteristics of participants are summarized in Table 1.

**Table 1:** Baseline Characteristics of Participants. Continuous variable age was presented as median (IQR) and categorical variables were presented as n and %.CPR: Cardiopulmonary Resuscitation; IQR: Interquartile Range;_____n: Number of participants; %: Percentage.

	**Types of Medical Professionals**					
	**Doctor (*N* = 36)**	**Nurse (*N* = 20)**	**Nurse Technician (*N* = 22)**	**Physicoterapist(*N* = 3)**	**Medical Students (*N* = 9)**	**Total (*N* = 90)**
Age, n (years)						
Median (IQR)	28.9 (27.3, 38.7)	28.7 (25.0, 34.2)	37.5 (27.2, 45.3)	51.3 (42.2, 54.7)	24.3 (23.4, 26.0)	29.1 (26.0, 39.2)
Sex, n (%)						
Male	21 (58.3 %)	6 (30.0 %)	12 (54.5 %)	2 (66.7 %)	2 (22.2 %)	43 (47.8 %)
Famale	15 (41.7 %)	14 (70.0 %)	10 (45.5 %)	1 (33.3 %)	7 (77.8 %)	47 (52.2 %)
Ethnicity, n (%)						
White	34 (94.4 %)	14 (70.0 %)	12 (54.5 %)	3 (100.0 %)	8 (88.9 %)	71 (78.9 %)
Black	0 (0.0 %)	1 (5.0 %)	3 (13.6 %)	0 (0.0 %)	0 (0.0 %)	4 (4.4 %)
Brown	0 (0.0 %)	4 (20.0 %)	7 (31.8 %)	0 (0.0 %)	1 (11.1 %)	12 (13.3 %)
Asian	2 (5.6 %)	1 (5.0 %)	0 (0.0 %)	0 (0.0 %)	0 (0.0 %)	3 (3.3 %)
CPR training level, n (%)						
Advanced life suppport	29 (80.6 %)	10 (50.0 %)	2 (9.1 %)	2 (66.7 %)	1 (11.1 %)	44 (48.9 %)
Basic life suppport	4 (11.1 %)	9 (45.0 %)	15 (68.2 %)	1 (33.3 %)	3 (33.3 %)	32 (35.6 %)
None	3 (8.3 %)	1 (5.0 %)	5 (22.7 %)	0 (0.0 %)	5 (55.6 %)	14 (15.6 %)
Last CPR Training, n (%)						
>2 years	11 (30.6 %)	2 (10.0 %)	3 (13.6 %)	0 (0.0 %)	2 (22.2 %)	18 (20.0 %)
<2 years	21 (58.3 %)	17 (85.0 %)	14 (63.6 %)	3 (100.0 %)	4 (44.4 %)	59 (65.6 %)
Not prior training	4 (11.1 %)	1 (5.0 %)	5 (22.7 %)	0 (0.0 %)	3 (33.3 %)	13 (14.4 %)
Perception of CPR quality metrics, n (%)						
Yes	35 (97.2 %)	20 (100.0 %)	19 (86.4 %)	3 (100.0 %)	8 (88.9 %)	85 (94.4 %)
No	1 (2.8 %)	0 (0.0 %)	3 (13.6 %)	0 (0.0 %)	1 (11.1 %)	5 (5.6 %)
Correct knowledge of compression depth, n (%)						
Yes	22 (61.1 %)	12 (60.0 %)	7 (31.8 %)	1 (33.3 %)	7 (77.8 %)	49 (54.4 %)
No	14 (38.9 %)	8 (40.0 %)	15 (68.2 %)	2 (66.7 %)	2 (22.2 %)	41 (45.6 %)
Correct knowledge of compression rate, n (%)						
Yes	31 (86.1 %)	18 (90.0 %)	12 (54.5 %)	3 (100.0 %)	8 (88.9 %)	72 (80.0 %)
No	5 (13.9 %)	2 (10.0 %)	10 (45.5 %)	0 (0.0 %)	1 (11.1 %)	18 (20.0 %)

**Fig. 2:**
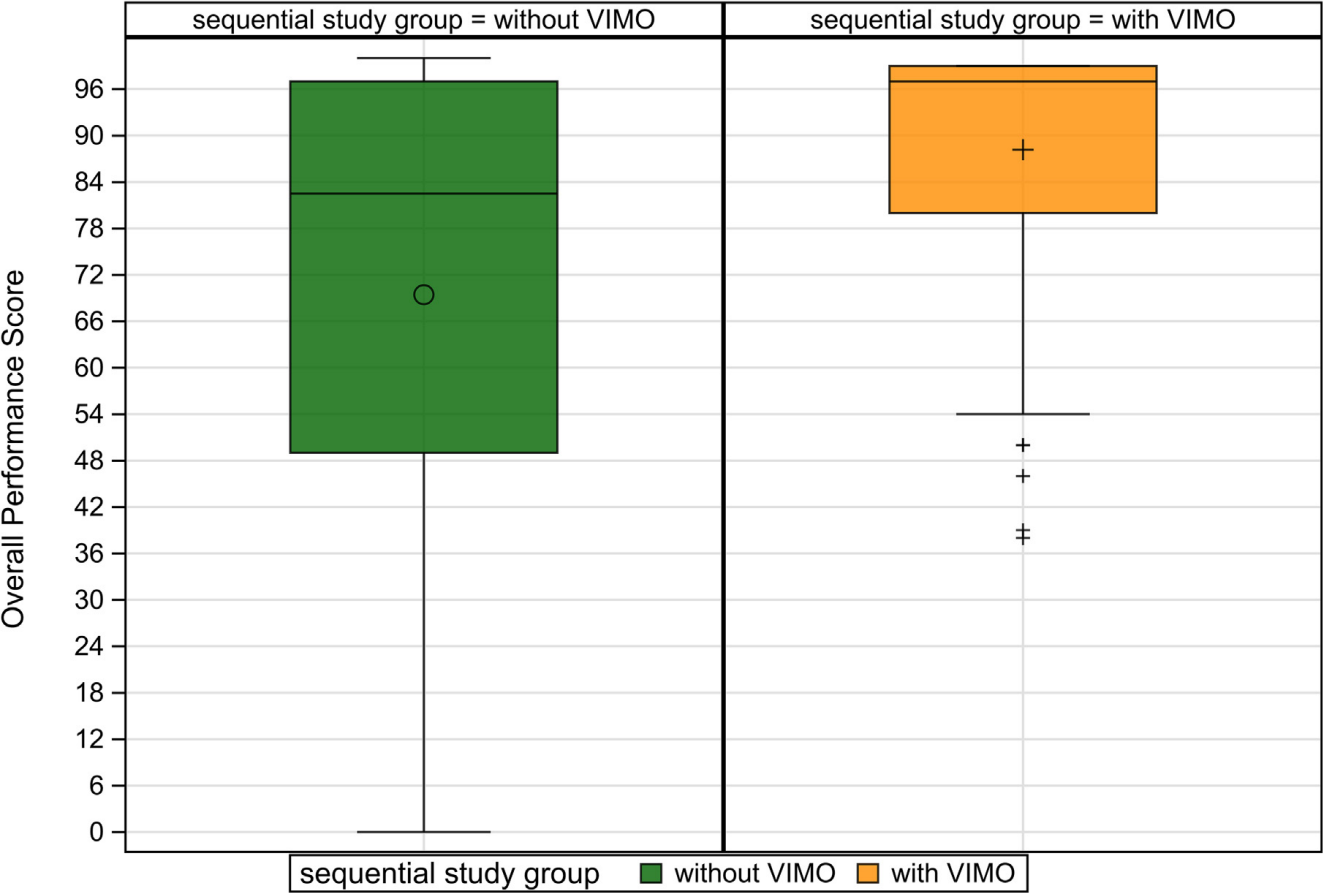
Overall performance score with and with smartwatch feedback.

A total of 90 healthcare professionals completed the simulation protocol. The median Overall Performance Score significantly improved with the use of the smartwatch feedback system, increasing from 82.5 % (IQR 49.0–97.0) without feedback to 97.0 % (IQR 80.0–99.0) with feedback (median difference: 19.5 (95 % CI: 13.5–27.5; *p* < 0.001) (Fig. 2).

**Fig. 3:**
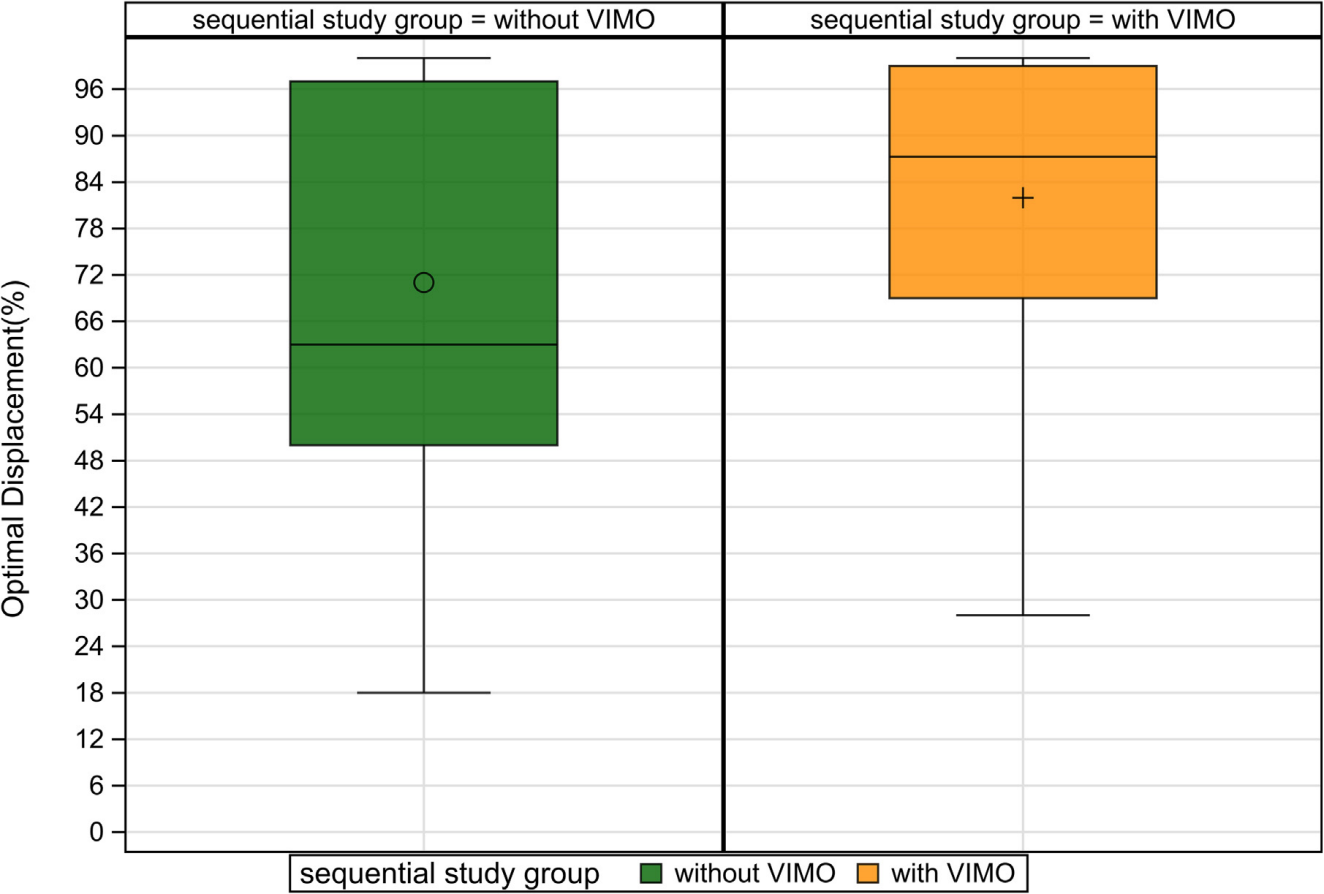
Optimal displacement with and without smartwatch feedback.

Among secondary outcomes, optimal displacement effectiveness improved from 71.0 % (95 % CI: 66.33–75.7) to 81.96 % (95 % CI: 78.1–85.8) with feedback (mean difference: 10.5 %; 95 % CI: 5.7–16.2; *p* < 0.001) (Fig. 3). The percentage of compressions within the correct rate range also increased substantially, from 62.5 % (IQR 15.0–94.0) to 94.5 % (IQR 70.0–100.0), with a median difference of 33.0 % (95 % CI: 24.0–42.5; *p* < 0.001) (Fig. 6).

**Fig. 4:**
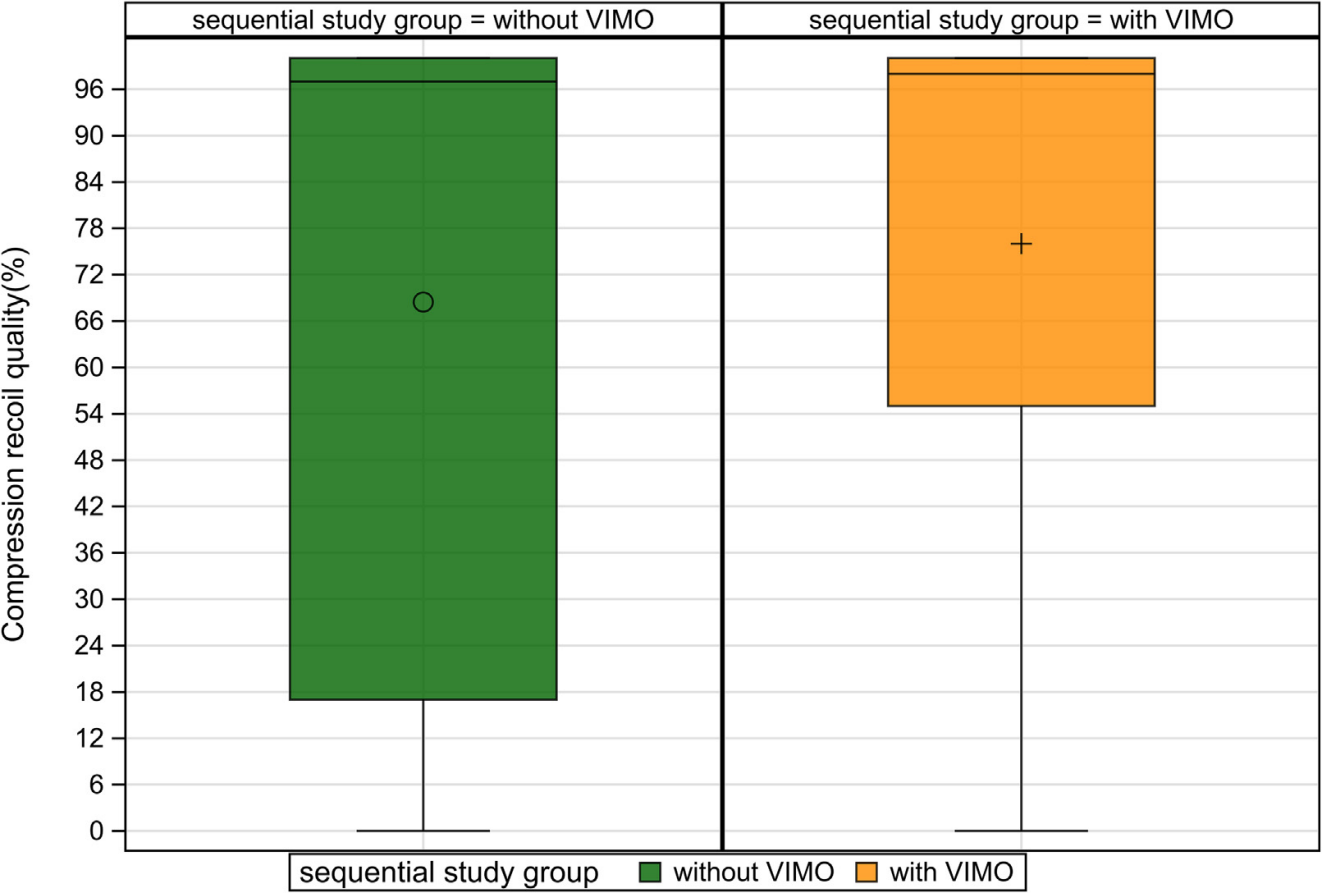
Compression recoil quality with and without smartwatch feedback.

**Fig. 5:**
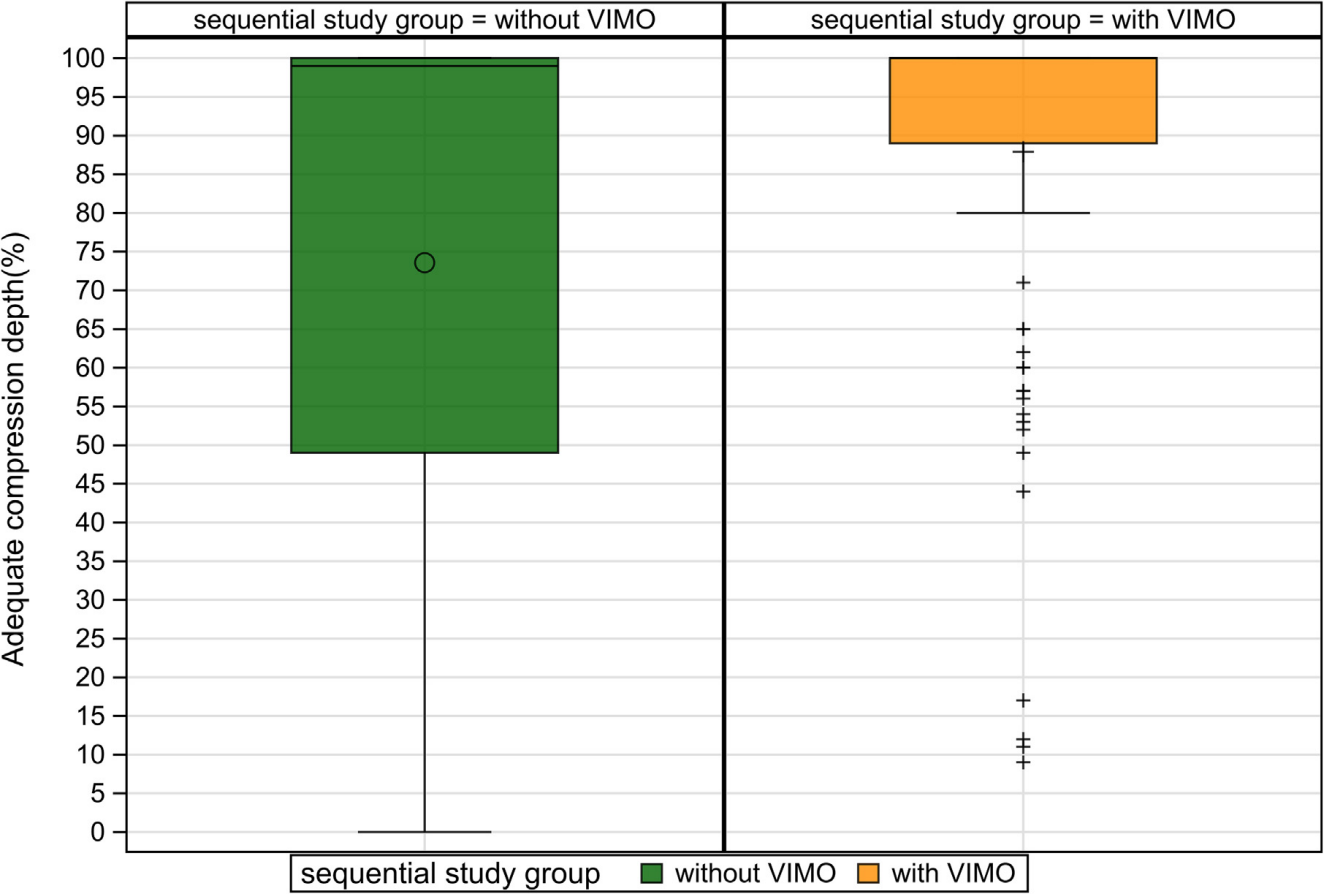
Adequate compression depth with and without smartwatch feedback.

**Fig. 6:**
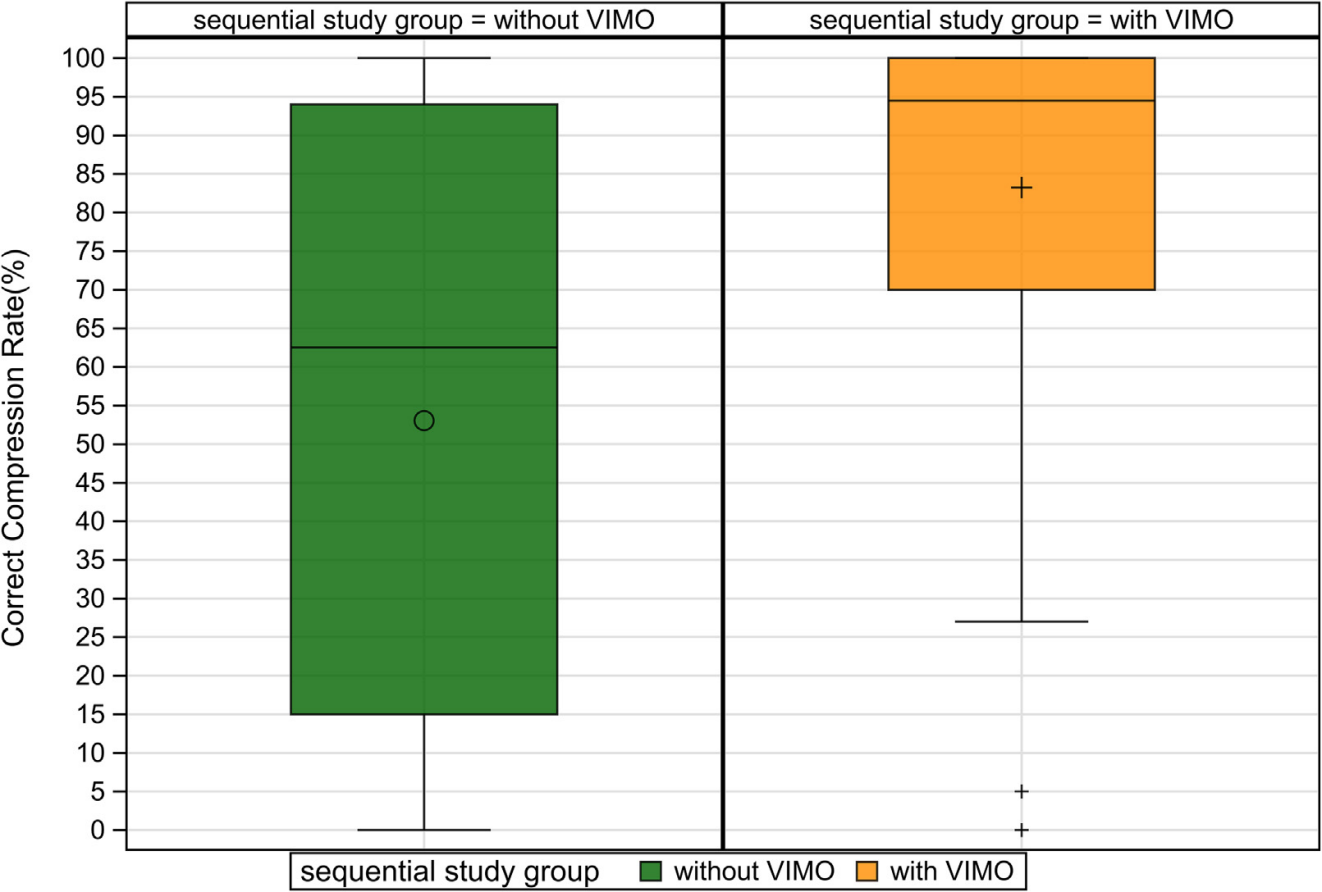
Correct compression rate with and without smartwatch feedback.

The percentage of adequate compression depth reached 100.0 % (IQR 89.0–100.0) with feedback versus 99.0 % (IQR 49.0–100.0) without, with a median difference of 23.5 % (95 % CI: 6.0–37.5; *p* = 0.002) (Fig. 5). Compression recoil quality showed no significant difference (median 98.0 % vs. 97.0 %; *p* = 0.080) (Fig. 4).

Other metrics such as average compression rate were slightly lower with feedback (without feedback median 116.0/min vs. with feedback median 112.0/min; *p* = 0.002) and the total number of compressions also decreased (without feedback median 229.0 vs. with feedback median 222.0; *p* = 0.006).

### Subgroup analyses

The subgroup analysis comparing the performance of participants categorized as doctors (*N* = 36) versus other healthcare professionals (*N* = 54) revealed no statistically significant differences across the evaluated metrics. Regarding the difference in Overall Performance Score, the median was similar in the group of other healthcare professionals (11.0; IQR: 1.0–44.0) (*N* = 36) compared to the doctors' group (8.0; IQR: 0.0–25.0), *p* = 0.322. For the difference in the percentage of optimal displacements, both groups showed similar medians: 7.3 (IQR: −0.3–34.3) for doctors and 6.8 (IQR: 0.0–26.5) for other healthcare professionals, *p* = 0.885. Regarding the percentage of correct compression rates, the median was not different among other healthcare professionals (27.5; IQR: 1.0–61.0) compared to doctors (14.5; IQR: 1.0–55.0), *p* = 0.742. Overall, although numerical differences were observed, none of the parameters demonstrated statistically significant differences. These findings highlight consistent performance across participant subgroups in this simulated resuscitation scenario.

The subgroup analysis based on CPR training level (Advanced, Basic, or None) revealed interesting variations in performance metrics. For the difference in Overall Performance Score, the median improvement was lowest in the Advanced group (7.5; IQR: 0.0–22.5), followed by the Basic group (11.5; IQR: 2.0–47.0), and highest in the group with no prior training (14.5; IQR: 2.0–64.0). Although there was a trend toward greater improvement among participants with no previous CPR training, this difference was not statistically significant (*p* = 0.083). For the difference in the proportion of optimal displacements, the medians were 5.3 (IQR: 0.0–32.8) in the Advanced group, 6.3 (IQR: 0.0–26.3) in the Basic group, and 15.8 (IQR: 0.0–36.0) in the group with no training, with no statistically significant differences between them (*p* = 0.728). However, the difference in the percentage of correct compression rate was statistically (*p* = 0.002): participants in the Basic group showed the greatest improvement (43.5; IQR: 16.5–77.0), followed by the group with no training (41.0; IQR: 14.0–64.0), while the Advanced group showed the smallest improvement (2.5; IQR: 0.0–31.0).

Following the significant KW test, the DSCF test was conducted for all possible pair comparisons between groups. The DSCF test showed significant pairwise comparisons between the following: Advanced vs basic (0.004) and Advanced vs No training (0.044). The comparison Basic vs No training was not significant (*p* = 0.834).

The subgroup analysis based on experience level (>2 years, <2 years, or “does not apply”) showed no statistically significant differences in performance improvements. Median differences in Overall Performance Score, proportion of optimal displacements, and percentage of correct compression rate.

## Discussion

This study demonstrates that the use of a feedback-enabled smartwatch significantly improves CPR quality across multiple performance metrics. The importance of high-quality CPR—defined by adequate depth, rate, full chest recoil, and minimal interruptions—is well established in international guidelines and supported by robust evidence.^16^ Nevertheless, real-world data consistently show suboptimal adherence to these targets, with inadequate compression depth observed in up to half of CPR attempts and compression rates frequently falling outside recommended ranges.[18], [36], [37], [38] Chest recoil, critical for diastolic filling and coronary perfusion, is also commonly neglected during decompression.^39^

These discrepancies arise from a combination of factors, including poor skill retention following training, cognitive overload during emergencies, and early onset of rescuer fatigue—often occurring within the first minute of CPR.[22], [40], [41] Feedback devices have emerged as valuable tools to mitigate these issues by providing real-time corrective guidance. Previous studies, including those using the CPR meter device (Laerdal/Philips), have demonstrated improvements in CPR performance in both training and clinical settings.^42^ Two important systematic reviews, realized by Yeung et al. and Lin et al., confirmed the benefit of such technologies in enhancing skill acquisition, though cost and limited accessibility restrict their widespread use.[16], [43]

Recent advances in wearable technology offer a promising alternative. Smartwatches equipped with motion sensors provide a practical, portable, and widely available solution for real-time CPR feedback. Our findings align with earlier simulation studies involving smartwatches by Gruenerbl et al., Ahn et al., and Lu et al., which showed improved compression rate and depth accuracy among laypersons and healthcare providers.[24], [25], [26]. Notably, Goharani et al. demonstrated improved clinical outcomes—including return of spontaneous circulation (ROSC) and survival to hospital discharge—in a real-life hospital-based trial using compression feedback assistance.^44^

In our study, participants demonstrated significantly improved Overall Performance Score with smartwatch feedback (97.0 % vs. 82.5 %; *p* < 0.001). Improvements were also observed in key secondary metrics, particularly compression rate (94.5 % vs. 62.5 %; *p* < 0.001) and optimal displacement (81.96 vs. 71.01, *p* < 0.001). These improvements suggest that real-time feedback helped participants maintain target parameters more consistently, even under simulated fatigue.

Although the difference in average compression rate between the feedback and no-feedback conditions reached statistical significance (without feedback median 116.0/min vs. with feedback median 112.0/min; *p* = 0.002), both median values remained within the guideline-recommended range of 100–120 compressions per minute. This difference of 4 compressions per minute is therefore unlikely to be clinically relevant, as prior evidence suggests that survival benefits are more sensitive to maintaining rates within the recommended range than to small variations within that range.

Notably, that full chest recoil was the only CPR quality metric that did not show significant improvement with smartwatch feedback. While this may reflect a true lack of effect, it is also possible that the wrist-worn accelerometer-based system used in this study was less sensitive or less accurate in detecting subtle variations in recoil compared to its validated performance in measuring compression rate and depth. Further external validation studies focusing specifically on recoil measurement accuracy are warranted.

One of the most notable improvements was the increase in the percentage of compressions delivered at the correct rate, which rose from 62.5 % to 94.5 % with the device (*p* < 0.001). This finding is particularly relevant, given that maintaining the recommended compression rate is crucial for achieving adequate coronary perfusion pressure and improving the likelihood of return of spontaneous circulation.

Additionally, the feedback system improved depth consistency. While the mean compression depth remained similar across conditions, the proportion of compressions that met the guideline-recommended depth threshold increased to 100 % with feedback (vs. 99.0 % without, *p* = 0.002). This suggests that real-time guidance helped participants apply more uniform and effective compression force, even under fatigue. These results underscore the value of continuous biomechanical monitoring in maintaining compression quality throughout the resuscitation effort.

Although the absolute difference in correct compression depth between scenarios was only 1 %, we believe this small improvement could be clinically relevant when extrapolated to real-world cardiac arrest situations. Previous studies have shown that even modest enhancements in CPR quality parameters, when sustained over time and across multiple rescuers, may contribute to improved physiological profiles and potentially better patient outcomes. Nevertheless, we recognize that the simulation-based design of our study limits direct extrapolation to clinical endpoints, and further research in real-world settings is warranted. It is notable that full chest recoil was the only CPR quality metric that did not show significant improvement with smartwatch feedback. While this may reflect a true lack of effect, it is also possible that the wrist-worn accelerometer-based system used in this study was less sensitive or less accurate in detecting subtle variations in recoil compared to its validated performance in measuring compression rate and depth.

On the other hand, compression recoil quality did not differ significantly between groups. This may be attributed to the participants’ high baseline performance in this metric or to technical limitations in the device’s capacity to detect incomplete recoil. Importantly, these findings were derived from a simulation-based study using manikins in a controlled environment. While this approach enables high internal validity, it does not fully replicate the complexities of real-life cardiac arrests, including patient variability, emotional stress, and environmental distractions. Therefore, caution is warranted when extrapolating these results to clinical settings. Additionally, the consistent magnitude of improvement observed across all subgroups, regardless of baseline CPR training or professional category, could be partially attributable to practice or familiarity effects inherent to the fixed-order design. Although the short 2-minute rest interval between trials was expected to attenuate such effects, it would not eliminate them. Because the study did not employ a randomized or counterbalanced order, we cannot fully separate the independent impact of the feedback device from potential learning bias. Future research should adopt a randomized crossover or counterbalanced design to more precisely isolate the effect of real-time feedback on CPR performance. While our findings demonstrate improvements in CPR quality metrics within a controlled simulation environment, several uncertainties limit direct extrapolation to clinical settings. Factors such as ambient noise, rescuer stress levels, variable patient chest compliance, and chaotic resuscitation environments may impair the usability and effectiveness of smartwatch feedback. Additionally, real-time audio prompts may be difficult to hear in noisy environments, and visual feedback may not be readily observable during active chest compressions. Integration with team leadership dynamics is another concern, as real-time feedback may either complement or conflict with verbal coaching provided by team leaders during resuscitations. Furthermore, the device’s performance has not been validated in patients with diverse body types or in scenarios involving prolonged resuscitation, where rescuer fatigue plays a more prominent role. Therefore, additional research in clinical or high-fidelity simulated environments that better replicate in-hospital and out-of-hospital cardiac arrest conditions are warranted before widespread clinical implementation.

The smartwatch provided real-time audio (metronome) and visual feedback during CPR. While participants could easily perceive both feedback modalities in the simulation setting, the feasibility of hearing the metronome sound in noisy clinical environments remains uncertain. Future development of alternative or additional feedback channels, such as haptic alerts or Bluetooth-linked external speakers, may enhance usability in real-world scenarios.

### Strengths and limitations

This study has several strengths, including its prospective paired design, which minimizes inter-participant variability, and the inclusion of a diverse sample of healthcare providers, enhancing generalizability. Additionally, the use of a next-generation smartwatch with real-time audio-visual feedback reflects a practical and scalable intervention with potential real-world applicability. Importantly, we propose the utilization of a wearable device that is already widely available on the wrists of the general population. By simply downloading the application, this smartwatch can be transformed into a CPR feedback device, enabling its use both for training purposes and in real-life emergency scenarios. This approach holds promise for increasing the likelihood of high-quality CPR delivery during out-of-hospital cardiac arrest events, potentially improving patient outcomes on a broader scale. Furthermore, the fixed-order design of this trial, combined with the short 2-minute interval between sessions, may have introduced participant fatigue, potentially attenuating the magnitude of the observed effects.

### Limitations

Study design: The non-randomized, fixed-order design introduces potential learning or warm-up effects that may have inflated the observed benefits. The absence of a counterbalanced or randomized sequence prevents definitive exclusion of this bias.
Primary outcome: The OPS, while a convenient summary of CPR quality, is an unvalidated composite metric, and its clinical significance for patient outcomes is unknown.
Setting and duration: The study was conducted in a controlled simulation environment over short intervals, limiting external validity and precluding assessment of clinical outcomes.
Statistical considerations: No formal multiplicity correction was applied despite multiple secondary and subgroup analyses, increasing the risk of Type I error. Subgroup analyses were exploratory, not pre-specified, and underpowered for detecting significant differences; thus, findings should be considered hypothesis-generating.
Measurement constraints: Smartwatch-based accelerometry may have reduced accuracy for certain parameters, such as complete chest recoil and hand positioning.

These limitations warrant cautious interpretation of the results and highlight the need for future randomized crossover studies and clinical trials to confirm the real-world effectiveness of smartwatch-guided CPR feedback systems.

### Implications and future directions

Given the widespread availability and affordability of consumer-grade smartwatches, this technology holds promise for use in out-of-hospital cardiac arrest (OHCA) scenarios and in low-resource settings where conventional CPR feedback devices are unavailable. For bystanders or lay rescuers, smartwatch-based feedback could offer accessible, real-time guidance to improve compression quality before emergency medical services arrive. In low-resource healthcare environments, where high-fidelity manikins or commercially available CPR feedback systems may not be feasible, smartwatches could serve as cost-effective training and real-time feedback tools. However, these potential applications require dedicated field studies to evaluate usability, accuracy, and impact on clinical outcomes in non-simulated, uncontrolled environments.

Although this study evaluated smartwatch feedback in healthcare professionals, the intuitive user interface and widespread availability of smartwatches suggest potential for adaptation to layperson use in the future. However, further usability testing, validation studies, and human factors research are needed to ensure the system’s effectiveness, safety, and user comprehension in non-professional populations.

Translating these findings to layperson and low-resource populations presents both opportunities and challenges. The widespread availability and intuitive interface of smartwatches could make this technology a scalable and low-cost tool to improve bystander CPR quality in OHCA scenarios. However, barriers such as the need for basic CPR training, environmental noise, and the lack of validation in untrained populations remain. Future research should focus on usability testing, real-world feasibility studies, and randomized trials in these target populations to assess clinical effectiveness and optimize implementation strategies.

While the observed improvements in CPR quality metrics are promising, this study was conducted in a manikin-based simulation environment and did not assess clinical outcomes such as return of spontaneous circulation, survival, or neurological recovery. Therefore, the clinical efficacy of smartwatch-based feedback systems remains unproven and requires validation in future human trials.

## Conclusion

In this simulation-based study involving healthcare professionals, the use of a smartwatch-based real-time feedback system significantly improved CPR compression rate and depth. However, caution is warranted when extrapolating these findings to real-world cardiac arrest scenarios. Further research is needed to validate these results in actual resuscitation environments and among laypersons.

### Ethical approval and informed consent

This study was approved by the Institutional Review Board of Hospital Alemão Oswaldo Cruz (Approval number: 2024-0801). All participants provided written informed consent before participation. The study was conducted in accordance with the Declaration of Helsinki and local regulatory standards.

We have also included this information in the Declarations section at the end of the manuscript, as per journal requirements.

## CRediT authorship contribution statement

**Leandro Menezes Alves da Costa:** Writing – review & editing, Writing – original draft, Validation, Methodology, Investigation, Formal analysis, Data curation, Conceptualization. **Rafael Otto Scheidewind:** Writing – review & editing, Writing – original draft, Validation, Methodology, Investigation, Data curation, Conceptualization. **Rogerio Ferrari Peron:** Conceptualization. **Thiago Timmerman:** Writing – review & editing, Writing – original draft, Validation, Methodology, Investigation, Data curation, Conceptualization. **Fabio de Cerqueira Lario:** Writing – review & editing, Writing – original draft, Validation, Supervision. **Frederico Rafael Moreira:** Writing – review & editing, Methodology, Formal analysis, Data curation, Conceptualization. **Rafael Amorim Belo Nunes:** Writing – review & editing, Methodology, Data curation, Conceptualization. **Thiago Luis Scudeler:** Writing – review & editing, Writing – original draft, Supervision, Methodology, Data curation, Conceptualization.

## Declaration of competing interest

The authors declare the following financial interests/personal relationships which may be considered as potential competing interests: Leandro Menezes Alves da Costa reports financial support was provided by VIMO SA. Leandro Menezes Alves da Costa reports a relationship with VIMO SA that includes: board membership and equity or stocks. Leandro Menezes Alves da Costa has patent pending to Licensee. No other other relationship or activity that may be interpreted as a conflict of interest by the reader. If there are other authors, they declare that they have no known competing financial interests or personal relationships that could have appeared to influence the work reported in this paper. Rogerio Ferrari Peron reports financial support was provided by VIMO SA. Rogerio Ferrari Peron reports a relationship with VIMO SA that includes: board membership and equity or stocks. Rogerio Ferrari Peron has patent pending to licensee. If there are other authors, they declare that they have no known competing financial interests or personal relationships that could have appeared to influence the work reported in this paper. Rafael Otto Scheneidewind reports financial support was provided by VIMO SA. Rafael Otto Scheneidewind reports a relationship with VIMO SA that includes: board membership and equity or stocks. Rafael Otto Schneidewind has patent pending to licensee. If there are other authors, they declare that they have no known competing financial interests or personal relationships that could have appeared to influence the work reported in this paper. Rafael Amorim Belo Nunes reports financial support was provided by VIMO SA. Rafael Amorim Belo Nunes reports a relationship with VIMO SA that includes: equity or stocks and non-financial support. If there are other authors, they declare that they have no known competing financial interests or personal relationships that could have appeared to influence the work reported in this paper. Thiago Luis Scudeler reports financial support was provided by VIMO SA. Thiago Luis Scudeler reports a relationship with VIMO SA that includes: equity or stocks and non-financial support. If there are other authors, they declare that they have no known competing financial interests or personal relationships that could have appeared to influence the work reported in this paper.
