# Supplementary material for: Effectiveness of a smartwatch-based feedback system in improving cardiopulmonary resuscitation quality: a simulation study
Source: Resusc Plus. 2025 Sep 30;26:101114. doi: 10.1016/j.resplu.2025.101114 (PMC12550195; doi:10.1016/j.resplu.2025.101114)
Supplement: Supplementary Data 8 [file mmc8.docx]

| **Variable name** | **Variable Label** | **Unit of measurement or code or format type** |
| --- | --- | --- |
| numero | Record ID | number |
| data_nascimento | date of birth | DD/MM/YYYY |
| Genero | Sex | 1 Male 2 Female |
| data_final | End date used in age calculation | End date used in age calculation  Age(in yrs) = (data_final-data_nascimento)/365.25 |
| Etnia | Ethnicity | 1=White  2=Black  3=Brown  4=Asian |
| pais_Nascimento | Country of birth | Brasil (1)= Brazil  Outro (2)= Other |
| formacao | Types of Medical Professionals | 1=Doctor  2=Nurse  3=Nurse Technician  4=Physicoterapist  5=Medical Students |
| Treinamento_RCP | CPR training level | 1=Advanced life support  2=Basic life support  3=None |
| ultimo_treinamento | Last CPR Training | 1: < 2 year  2: >= 2 years  3: no training |
| metricas | Perception of CPR quality metrics | 1=Yes  2=No |
| Profundidade | Correct knowledge of compression depth | 1=Yes  2=No |
| Frequencia | Correct knowledge of compression rate | 1=Yes  2=No |
| Pontuacao_Total_1 | Overall Performance Score without VIMO | Numeric |
| Pontuacao_Total_2 | Overall Performance Score with VIMO | Numeric |
| Total_Compressoes_1 | Total number of compressions without VIMO | Numeric |
| Total_Compressoes_2 | Total number of compressions with VIMO | Numeric |
| Boa_liberacao_1 | Compression recoil quality without VIMO | Percentage (%) |
| Boa_liberacao_2 | Compression recoil quality with VIMO | Percentage (%) |
| Boa_profundidade_1 | Adequate compression depth without VIMO | Percentage (%) |
| Boa_profundidade_2 | Adequate compression depth with VIMO | Percentage (%) |
| pct_FC_Correta_1 | Correct Compression Rate without VIMO | Percentage (%) |
| pct_FC_Correta_2 | Correct Compression Rate with VIMO | Percentage (%) |
| FC_media_1 | Average compression rate without VIMO | Rate /minute |
| FC_media_2 | Average compression rate with VIMO | Rate /minute |
| Fracao_compressao_1 | Compression fraction without VIMO | Percentage (%) |
| Fracao_compressao_2 | compression fraction with VIMO | Percentage (%) |
| Profundidade_media_1 | Average depth without VIMO | Numeric in millimeters |
| Profundidade_media_2 | Average depth with VIMO | Numeric in millimeters |
| Deslocamento_otimo_1 | Optimal displacement without VIMO | Percentage (%) |
| Deslocamento_otimo_2 | Optimal displacement with VIMO | Percentage (%) |
