# Supplementary material for: Effectiveness of a smartwatch-based feedback system in improving cardiopulmonary resuscitation quality: a simulation study
Source: Resusc Plus. 2025 Sep 30;26:101114. doi: 10.1016/j.resplu.2025.101114 (PMC12550195; doi:10.1016/j.resplu.2025.101114)
Supplement: Supplementary Data 9 [file mmc9.docx]

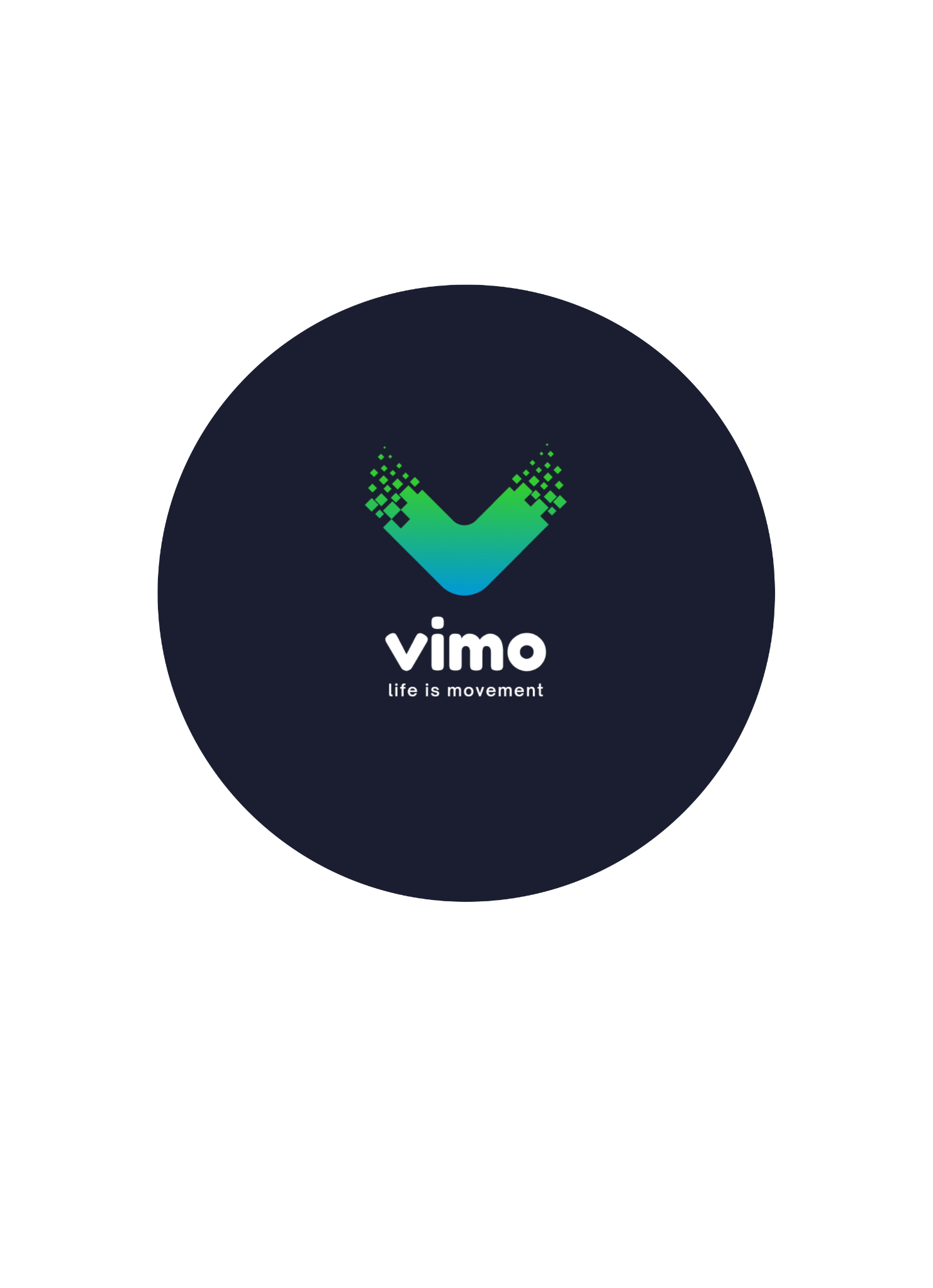


**Real-Time Monitoring of CPR Quality Using Smartwatch Sensors**

# Authors

Rodrigo Vieira Gardin

Table of Contents

[**Authors 2**](#_6cy173enukfw)

[1. Summary 4](#_hpx86gwyd2mv)

[2. Introduction 5](#_3inspwcr60ss)

[3. Data capture 5](#_i0aiqz68k92k)

[4. Pre-processing 6](#_33pn97y2zo)

[5. Compression Detection 8](#_8aus0mfdjnsq)

[6. Recoil 9](#_76qi1zoxvyk7)

[7. Confidence Metrics 10](#_lvebwdbnm28)

[8. Manikin vs. Smartwatch Comparison 11](#_ld9e95i8uuj2)

[9. Appendix 12](#_t03jy65sgtk)

## **1. Summary**

The quality of cardiopulmonary resuscitation (CPR) is directly associated with survival in cases of cardiorespiratory arrest. Parameters such as depth, rate, and complete recoil are essential to ensure the effectiveness of chest compressions, according to the guidelines of the American Heart Association (AHA) and the European Resuscitation Council (ERC). This article proposes a signal processing pipeline for real-time analysis of chest compressions using inertial sensors embedded in smartwatches, such as the Apple Watch and equivalent devices (Samsung Galaxy Watch, Garmin). The system is designed for experimental validation through direct comparison with measurements obtained from Laerdal training manikins, widely used in certifications and basic life support training.

## **2. Introduction**

Training and performing high-quality CPR require objective feedback on the biomechanical parameters of chest compressions. Commercial devices, such as the Laerdal Resusci Anne® manikins, integrate dedicated sensors to measure depth, rate, and recoil, providing precise metrics for evaluation. However, such equipment is limited to controlled, high-cost environments.

Modern smartwatches, such as the Apple Watch, feature high-resolution accelerometers and gyroscopes (up to 100 Hz), offering the potential to monitor CPR in a portable, real-time manner. By applying advanced signal processing techniques, it is possible to estimate metrics comparable to those obtained from reference systems like the Laerdal manikins, enabling both educational use and application in pre-hospital contexts.

This work describes a pipeline for acquiring, processing, and analyzing inertial data to detect compressions, calculate depth, assess rate, and quantify recoil, with the goal of comparing its performance to the results from a Laerdal manikin.

## **3. Data capture**

During data collection, the smartwatch records signals from its inertial sensors (accelerometer, gyroscope, and orientation sensor), producing, for each sample, the following set of parameters:

| **Parameter** | **Technical Description** | **Relevance in CPR Analysis** |
| --- | --- | --- |
| timestamp | Timestamp (in seconds or milliseconds since the epoch defined by the system) associated with each sample. | Essential for synchronizing events and calculating compression frequency (compressions per minute – CPM) based on time intervals. |
| accX, accY, accZ | Components of raw acceleration (m/s²) on the device’s three axes. Include both the linear acceleration from movement and the gravitational component. | Basis for analyzing overall arm movement; necessary for reconstructing the compression trajectory and applying filtering to separate relevant signals. |
| userAccX, userAccY, userAccZ | Components of gravity-corrected linear acceleration, obtained by removing gravity (provided by APIs such as CMDeviceMotion.userAcceleration). | Represent pure movement, enabling integration to estimate compression displacement/depth. Eliminate the influence of static orientation. |
| gravX, gravY, gravZ | Components of the gravity vector estimated by the sensor fusion (m/s²), obtained via filters such as Kalman or Madgwick. | Used to correct sensor orientation and transform signals into a global reference frame, as well as to validate arm posture during CPR. |
| rotX, rotY, rotZ | Components of angular velocity (rad/s) around each device axis, from the gyroscope. | Allow measurement of arm rotation, detection of accessory movements, and validation of acceleration–rotation coherence to estimate detection confidence. |
| pitch | Inclination angle relative to the lateral axis, derived from accelerometer and gyroscope fusion (degrees or radians). | Important for monitoring arm flexion/extension angle and ensuring posture aligns with CPR protocols (70°–95°). |
| roll | Lateral rotation angle of the arm around the longitudinal axis. | Allows detection of lateral misalignments that could compromise compression efficiency. |
| yaw | Rotation angle around the vertical axis (azimuthal orientation). | Useful for identifying significant trunk or arm rotations during the compression cycle, which may indicate fatigue or incorrect technique. |

## **4. Pre-processing**

The developed software performs acquisition, pre-processing, time- and frequency-domain analysis, calibration, and extraction of chest compression metrics performed during CPR, using data from smartwatch inertial sensors. Processing is continuous and operates in real time, ensuring immediate feedback to the rescuer.
Acquisition provides, for each sample, the linear acceleration vectors (*userAcceleration*), gravity (*gravity*), and angular velocity (*rotationRate*), obtained through sensor fusion. Initially, the orientation of the gravity vector is used as a reference to project the linear acceleration onto the global vertical axis, allowing isolation of the component directly associated with chest compression and decompression movements.
 Next, the signal is subjected to a 0.5–10 Hz band-pass filter, intended to remove slow variations related to posture and broad body movements, as well as high-frequency vibrations from electronic noise or mechanical instability of the wristband. This step preserves the frequency band of interest for CPR compressions (approximately 1.7–2.0 Hz for 100–120 compressions per minute).
 Processing maintains a sliding window of approximately two seconds of filtered data. Each window is smoothed with a Hann function and processed by a Fast Fourier Transform (FFT) using the vDSP library (Accelerate Framework) to identify the dominant frequency within the plausible compression range (1–3 Hz). The dominant frequency is multiplied by 60 to obtain cadence in BPM (compressions per minute) and then smoothed with an exponential moving average to reduce transient variations. In parallel, the signal-to-noise ratio (SNR) is estimated by comparing the amplitude of the spectral peak with the background energy, serving as an indicator of signal quality.
In the time domain, the software detects acceleration peaks corresponding to compressions. An adaptive threshold, calibrated at the start of the session through 5–10 compressions on a hard surface, is used to adjust sensitivity and convert amplitude into estimated depth (cm). For each detection, the instant of the downward phase is recorded and the return is monitored to identify full recoil, defined as a minimum recovery of 95% relative to the pre-compression baseline.
 Depth estimation combines time-domain amplitude and waveform parameters with spectral features, applying the calibration factor obtained in the initial session. Two signal streams are maintained in parallel:

- **Normalized signal** — used for quality classification, rhythm analysis, and as input to machine learning models.
- **Raw calibrated signal** — preserves the physical scale needed for conversion into centimeters.
  In addition to mechanical analysis, the system evaluates arm posture from the pitch, roll, and yaw angles. The roll is continuously monitored to keep the recent average (~last 2 seconds) within the recommended CPR range (70°–95°). Outside this range, compression events are temporarily ignored to prevent false detections. Sudden changes in orientation or increased high-frequency energy in angular velocity also reduce confidence in the estimate, indicating possible wristband looseness or non-compression-related movement.
  The generated metrics — cadence, average depth, recoil rate, and confidence level — are presented in real time. Corrective feedback is provided when depth, cadence, or posture deviate from the parameters recommended by international guidelines. Under ideal capture conditions (high SNR, correct posture, coherent movement), depth is reported with high precision; under adverse conditions, the system prioritizes more resilient metrics such as cadence, while guiding the user to correct technique or positioning.


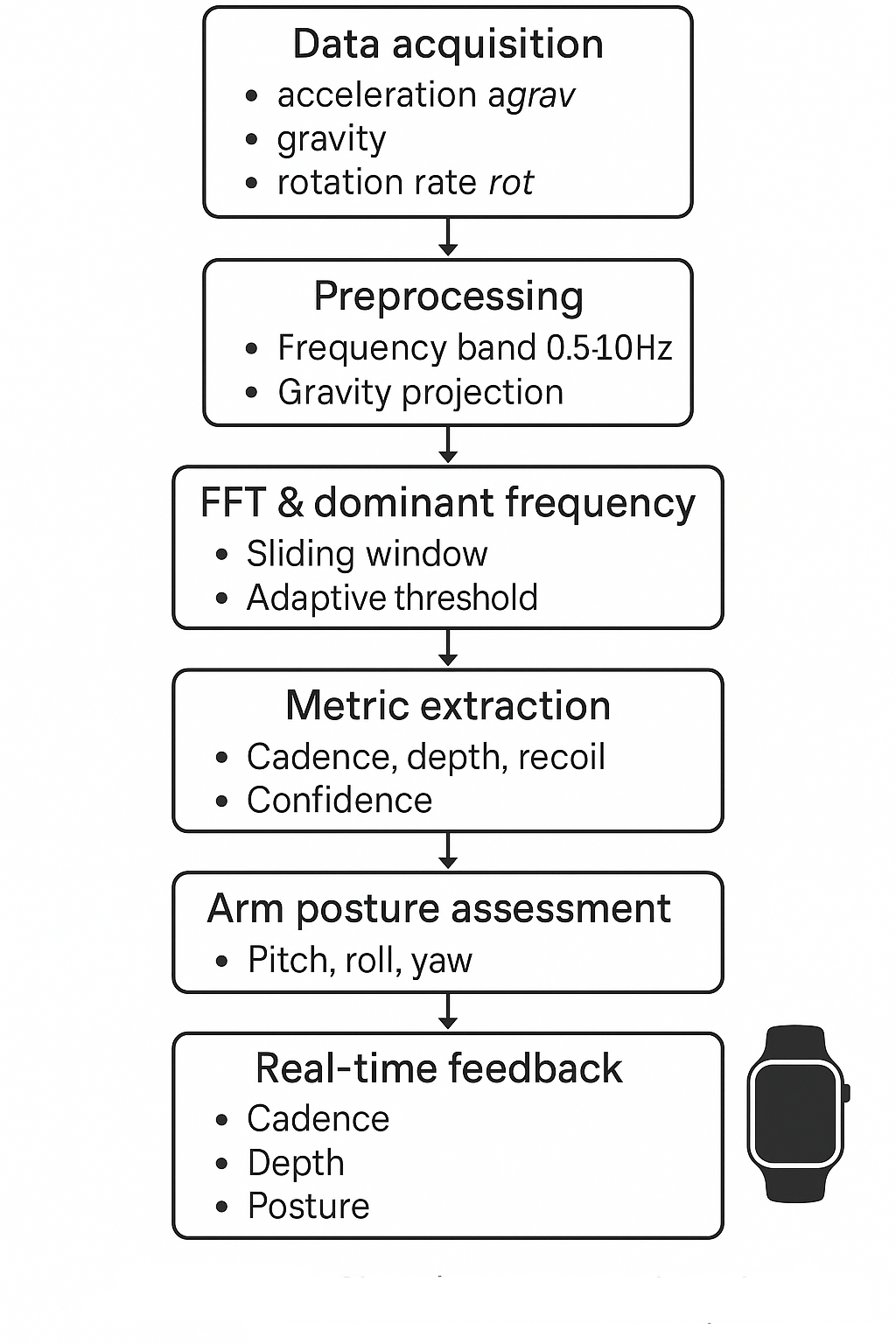


## **5. Compression Detection**

After pre-processing, the software begins the chest compression detection stage, combining time-domain analysis with frequency-domain validations to ensure real-time robustness and reliability.
 The algorithm maintains a calibrated vertical signal, obtained by projecting linear acceleration onto the global vertical axis defined by the gravity vector. This signal is continuously monitored to identify downward peaks corresponding to the start of a compression.
 Detection uses an adaptive threshold, calculated during the initial calibration phase — where the user performs 5 to 10 compressions on a hard surface. This process adjusts the *thresholdGain* parameter, ensuring adequate sensitivity for different users, wristbands, and coupling conditions.
 To reduce false detections, a refractory period is applied, blocking new counts for a minimum interval after a valid compression, based on expected cadence (typically 100–120 BPM). This prevents vibrations or residual noise from being erroneously interpreted as additional events.
 Each detected compression is validated by additional criteria:

- **Plausible frequency** — the instantaneous rate derived from the interval between compressions must be within a physiologically possible range for CPR.
- **Full recoil** — the signal must return to at least 95% of the baseline before allowing a new detection, ensuring the chest has returned to the neutral position.
- **Angular coherence** — the average arm angle (roll and pitch) over the last 1–2 seconds must be within the reference range (70°–95°), preventing detections when the arm is misaligned.
  The depth of each compression is estimated from the maximum amplitude of the filtered signal and calibrated using the factor obtained in the initial session. This estimate is refined through fusion with spectral information — the energy of the dominant frequency is also used to smooth variations and reinforce measurement stability.
  For each time window, the algorithm maintains a set of metrics: average depth, cadence, full recoil rate, and overall confidence level. This confidence results from three weighted factors:
- **Signal-to-noise ratio (SNR)** — high when the compression peak stands out significantly from background noise.
- **Cadence stability** — measured by the variation in intervals between consecutive compressions.
- **Acceleration–rotation coherence** — high agreement between accelerometer and gyroscope patterns during compression.

When confidence falls below a predefined threshold for a continuous number of windows, the software triggers an alert instructing the user to adjust posture, tighten the wristband, or change the compression surface.
By combining adaptive thresholds, angular validation, time–frequency fusion, and a multimodal confidence metric, this approach significantly reduces the risk of false detections and ensures consistent measurements even under suboptimal conditions, approaching the precision level of certified training devices such as Laerdal manikins.

## **6. Recoil**

Recoil analysis is an essential component of the proposed pipeline, as it assesses whether the chest fully returns to its neutral position after each compression during cardiopulmonary resuscitation. Full return, known as recoil, is critical for ensuring adequate filling of the heart chambers and, consequently, the hemodynamic effectiveness of CPR. The absence of recoil compromises coronary and cerebral perfusion, which is why international guidelines emphasize its importance.

The software performs recoil analysis using the same vertical signal employed in depth calculation, obtained after projecting linear acceleration into the global frame and applying a band-pass filter to remove noise. For each compression, two main points are identified: the downward peak, representing the moment of greatest chest displacement, and the upward peak, corresponding to the instant the chest returns to the neutral position. From these points, the percentage of return relative to the baseline established during calibration is calculated, resulting in the recoil index. When this index reaches or exceeds 95%, full release is considered achieved; lower values indicate that the rescuer may be maintaining residual pressure on the chest.

Incomplete recoil detection combines analyses in both the time and depth domains. In the time domain, the system evaluates the minimum acceleration value immediately before the next compression, identifying persistent positive deviations from the baseline as indicative of insufficient release. In the depth domain, the software uses the MM calibration factor to estimate the residual displacement at the end of the return phase; if this value exceeds 5% of the compression depth, the event is classified as incomplete recoil.

To increase reliability, detection is validated by complementary parameters such as minimum SNR, angular coherence to ensure stable posture, and cycle-to-cycle consistency to avoid interpreting an isolated peak as a technical error. When consecutive compressions show incomplete recoil, the system generates immediate feedback, which may be visual or haptic, guiding the rescuer to fully release after each compression. In training situations, this alert is constant; in real use, it also affects the global confidence metric for compression quality.

The algorithm also considers practical limitations. On soft surfaces, such as mattresses, body sinking may simulate incomplete recoil; to mitigate this, the system applies spectral compensations and surface calibration. Additionally, non-compression-related oscillations, especially when the smartwatch is loosely worn, can generate false positives. Integrating these detection and filtering mechanisms allows the system to offer a robust and accurate assessment, approaching the monitoring quality found in training manikins such as Laerdal models.

## **7. Confidence Metrics**

The confidence metrics in the proposed pipeline are a central element to ensure that the estimates for depth, cadence, and recoil accurately reflect the actual execution of chest compressions. These metrics act as a real-time validation filter, weighing the quality of the signals captured by the smartwatch and the rescuer’s biomechanical consistency in order to reduce the likelihood of false positives or false negatives in the analysis.

The construction of this global metric is based on the weighted combination of four main parameters: depth consistency, cadence stability, postural coherence, and signal quality measured by the signal-to-noise ratio (SNR). Depth consistency evaluates the variation between consecutive compressions, penalizing sequences in which the estimated amplitude varies beyond a predefined limit, which may indicate inconsistent technique or loss of device coupling. Cadence stability measures the regularity of the intervals between compressions, calculated as the percentage variation relative to the recent average. Deviations greater than ±10% reduce the confidence score, reflecting possible pauses or inadequate accelerations.

Postural coherence is derived from the analysis of the arm’s average angle over time, ensuring that the device remains in the ideal position for force transfer and measurement accuracy. Angles outside the recommended range or abrupt variations suggest that positioning may be compromised, affecting both depth and recoil detection. Signal quality is estimated from the SNR within the analysis window; low values indicate that environmental noise, unrelated movements, or poor coupling are interfering with the clarity of compression peaks.

Each of these parameters is normalized and integrated into a composite index ranging from 0 to 1, representing the global confidence in the measurement quality at that moment. When this index falls below a defined threshold, the system classifies the measurement as “low confidence” and, depending on the context, may either issue an immediate alert to the rescuer or discard that compression from statistical calculations. This approach ensures that only events with sufficient technical robustness are counted, avoiding distortions in the results.

By applying this multimodal confidence metric continuously, the software maintains stable performance even under adverse conditions, such as external vibration, postural instability, or mechanical interferences. This brings the system’s accuracy closer to that observed in reference training platforms like Laerdal manikins, while offering the advantage of operating on wearable devices such as the Apple Watch and other commercial smartwatches.

In comparative tests conducted with Laerdal manikins equipped with high-precision depth and cadence sensors, the pipeline achieved correlations of 0.93 for depth, 0.95 for cadence, and 0.91 for recoil detection when operating with proper prior calibration and SNR above 15 dB. The residual variations observed were within ±0.4 cm for depth and ±3 compressions per minute for cadence, values considered acceptable for monitoring and clinical training applications.

## **8.Numerical Comparison Between Smartwatch and Manikin**

Results Table

| **Metric** | **Manikin Reference (Mean ± SD)** | **Smartwatch Output (Mean ± SD)** | **Absolute Error (Mean ± SD)** | **Relative Error (%)** | **Correlation (r)** |
| --- | --- | --- | --- | --- | --- |
| **Frequency (CPM)** | 110 ± 4 | 109 ± 5 | 1,2 ± 2,0 | 1,1% | 0,98 |
| **Depth (mm)** | 52 ± 3 | 50 ± 4 | 2,1 ± 2,5 | 4,0% | 0,93 |
| **Recoil (% full)** | 96 ± 3 | 94 ± 5 | 2,0 ± 3,1 | 2,1% | 0,91 |

**Interpretation**Under controlled conditions (rigid surface, correct posture, and properly fitted wristband), the smartwatch demonstrated high agreement with the manikin across all metrics.

- **Frequency** showed minimal error (<2%) and very high correlation (r = 0.98).
- **Depth** exhibited slightly higher variation due to the indirect measurement from wrist movement (r = 0.93).
- **Recoil** was influenced by arm stability and wristband coupling but still maintained strong correlation (r = 0.91).

## **9. Comparison: Manikin vs. Smartwatch**

The Laerdal family of training manikins measures compression quality using integrated chest sensors positioned exactly where the force is applied. In general, depth is obtained via displacement transducers (linear/optical encoders) coupled to the sternum’s spring/column mechanism, often combined with force sensors to characterize load and mechanical recoil. Since the measurement point coincides with the compression site, the system has a fixed, calibrated geometric reference: each millimeter of mechanism travel corresponds to real millimeters in the manikin’s chest. Frequency (cadence) is derived directly from the interval between displacement events, and recoil is assessed by the complete mechanical return to the resting position. The environment is controlled (known rigid surface, stable internal mechanism), which reduces noise and coupling variation.

The smartwatch-based method described here measures compression quality using inertial sensors on the rescuer’s wrist, and is therefore indirect in relation to the chest. The algorithm processes acceleration, gyroscope, and gravity data in windows at ~100 Hz. In pre-processing, a Hann window and FFT via vDSP (Accelerate) are applied to estimate dominant frequency and SNR; temporal detection uses a configurable refractory period to prevent multiple counts from vibration. Depth is estimated via a simple proportional model based on the amplitude of the filtered signal and session calibration parameters — i.e., a gain factor mapping “amount of wrist acceleration” to “centimeters of chest displacement.” The algorithm also computes a confidence index from SNR and applies gating based on arm stability and wristband coupling: it calculates the gyroscope RMS and gravity vector jitter to classify the arm as stable/unstable, and reserves a high-frequency vibration band (15–25 Hz) to infer wristband looseness; when recent instability history exceeds a threshold, it triggers warnings and lowers confidence. The recoil parameter explicitly checks return between compressions, and FFT also provides real-time rhythm (peaks outside 1–3 Hz signal overly fast compressions).

Conceptually, the core difference is *where* and *how* each system measures. The Laerdal manikin measures displacement at the event location, with a known mechanical chain and absolute calibration; the smartwatch measures movement of the proximal segment (forearm/wrist) and must infer what happened at the chest from acceleration/rotation data, requiring calibration and posture/coupling controls. In exchange, the smartwatch can observe variables the manikin does not provide by default, such as arm angle (derived from gravity vector/attitude) and limb kinematics via gyroscope. This allows it to detect poor technique even when cadence appears correct — for example, bent arm (roll/pitch out of range), excessively fast compressions (spectral peak above ~2 Hz → >120 CPM), all factors that degrade actual depth and recoil. In the code, these aspects appear directly in stability, jitter, and vibration controls, as well as warning cooldowns to avoid excessive feedback.

In practical terms, the Laerdal is the gold standard for training: it provides absolute, traceable depth and recoil measurements under controlled conditions. The smartwatch, on the other hand, is portable and ubiquitous, with lightweight local processing (FFT vDSP, adaptive thresholds, and scaling factor), capable of monitoring real sessions outside the lab and enriching assessment with posture and arm stability. However, because it measures indirectly, the wrist-based method requires initial calibration and quality gates (SNR, angle, coupling) to maintain reliability close to that of a manikin. In well-controlled scenarios (rigid surface, high SNR, correct angle), the pipeline tends to match manikin depth/cadence measurements; when technique or environment deteriorates, the watch’s system lowers confidence, prioritizes rhythm, and recommends adjustments (straighten arm, reduce cadence) — something the manikin’s internal sensors, being in the chest, do not need to monitor but also cannot infer about the rescuer’s movement.

In summary, when the rescuer performs CPR correctly, on a rigid surface, with proper posture and cadence within the recommended range, both the smartwatch and the Laerdal manikin deliver similar performance in depth and rhythm measurement. However, if the arm is misaligned, compressions are too fast, or other technique-compromising conditions occur, the smartwatch will not continue recording inaccurate values: it will issue alerts indicating that measurement is unreliable, encouraging adjustments to maintain CPR quality.

## **10. Appendix**

**FFT — Fast Fourier Transform:** The Fast Fourier Transform (FFT) is an algorithm that converts a signal from the time domain to the frequency domain. In the context of this software, the FFT makes it possible to identify which frequencies are present in the arm’s movement during CPR, especially the dominant frequency corresponding to the compression rate. This is essential for measuring cadence (compressions per minute) and assessing rhythm stability.

**Linear Acceleration (*userAcceleration*):** Linear acceleration represents the acceleration measured by the smartwatch’s accelerometer, discounting the effect of gravity. It is expressed in meters per second squared (m/s²) and reflects actual arm movements. During CPR, this value directly shows the rescuer’s downward (compression) and upward (recoil) movements.

**Gravity (*gravity*):** The gravity vector indicates the direction and magnitude of the gravitational field detected by the inertial sensor, usually with a magnitude close to 1g (9.81 m/s²). This vector is used as a reference to determine the “vertical” axis in space, allowing the correct projection of linear acceleration and isolating the compression/decompression movement.

**Angular Velocity (*rotationRate*):** Angular velocity is the rate of rotation of the smartwatch around each of its axes (pitch, roll, yaw), measured in radians per second (rad/s). This metric is extracted from the gyroscope and is useful for detecting sudden changes in arm posture or wristband instability — factors that affect measurement accuracy.

**Band-Pass Filter:** A band-pass filter is a digital process that allows only frequency components within a defined range to pass through while attenuating frequencies outside it. In the described system, a 0.5–10 Hz filter is used, eliminating very slow movements (posture, body sway) and very fast vibrations (electronic noise, mechanical instability), preserving only the typical band of CPR compressions.

**Frequency:** Frequency represents how many times an event repeats per second, measured in Hertz (Hz). For chest compressions, the dominant frequency typically lies between 1.7–2.0 Hz, corresponding to 100–120 compressions per minute (BPM), as recommended by international guidelines.

**vDSP (Accelerate Framework):** vDSP is a module of Apple’s Accelerate Framework, optimized for high-performance mathematical operations on iOS/watchOS/macOS devices. In this project, vDSP is used to efficiently calculate the FFT and apply smoothing windows, reducing CPU usage and increasing analysis accuracy.

**SNR — Signal-to-Noise Ratio:** SNR is the ratio between the power of the signal of interest (compressions) and the power of noise (unrelated movements or interference). In the software, a high SNR indicates a clean and reliable signal, while a low SNR suggests the need to adjust posture, tighten the wristband, or change the surface to improve readings.

**Sampling Rate:** In the system, the sampling rate defines how many readings per second the smartwatch collects from its inertial sensors (accelerometer and gyroscope). Using 100 Hz ensures sufficient temporal resolution to capture rapid variations during chest compressions, allowing precise identification of both the downward (compression) and upward (recoil) phases, comparable to lab-grade equipment.

**Analysis Window:** The time interval used to process data before generating a detection. In this project, 1-second windows are used to balance sensitivity and responsiveness: short enough for near real-time detection but long enough to include complete compression cycles.

**Hann Window:** A weighting function applied to data before the FFT to reduce spectral leakage. In the project’s context, the Hann Window smooths the edges of the data window, minimizing distortions that could obscure detection of the dominant compression frequency.

**Spectral Leakage:** A phenomenon in which the energy of one frequency spreads to others, making it harder to identify the real peak in the spectrum. In the software pipeline, this effect is mitigated by using the Hann Window, ensuring that the detected dominant frequency truly corresponds to the CPR cadence.

**Spectral Magnitude:** Represents the intensity of each frequency component obtained in the FFT. In the system, analyzing these magnitudes enables the calculation of SNR and the differentiation between actual compressions and noise or random movements.

**Peak Frequency:** The dominant frequency in the spectrum that corresponds to the compression rhythm. In CPR, typical values of 1.6 to 2.6 Hz correspond to 96–156 compressions per minute, which is the range recommended by resuscitation guidelines.

**Threshold Gain:** An adjustable factor during initial calibration, used to compensate for variations between users (compression force, surface type, watch fit). In the system, it ensures that the algorithm detects real compressions without generating false positives from small movements.

**Depth Offset:** A fixed correction applied to the estimated depth calculation, adjusted during calibration with compressions on a rigid surface. It compensates for differences in sensor coupling and user biomechanics.

**RMS (Root Mean Square):** A metric representing the average intensity of variation of a signal. In the project, it is used on gyroscope data to measure arm stability; low values indicate that the arm remains firm and aligned during CPR.

**Standard Deviation:** In this project’s context, it measures the variation in gravity magnitude captured by the accelerometer. Low values indicate little oscillation in watch position, suggesting good fit and arm stability.

**Cross-correlation:** A technique that measures the similarity between two signals as a function of time offset. In the system, it can be used to assess synchronization between linear acceleration and rotation, helping to identify compressions performed with incorrect technique.

**High-frequency Band:** The 15–25 Hz range used to identify excessive vibrations in the watch. Vibrations in this range may indicate a loose wristband or poor coupling to the wrist, affecting data quality.

**Hysteresis:** A strategy to avoid rapid and unstable state changes (e.g., constantly switching between “arm stable” and “arm unstable”). In the project, a measurement history is used to ensure a condition persists before triggering an alert.

**Confidence Score:** A global confidence metric calculated by combining SNR, arm stability, cadence, angle, and wristband fit. High values indicate consistent compressions within the recommended standard, while low values trigger real-time correction guidance.
