# Supplementary material for: Effectiveness of a smartwatch-based feedback system in improving cardiopulmonary resuscitation quality: a simulation study
Source: Resusc Plus. 2025 Sep 30;26:101114. doi: 10.1016/j.resplu.2025.101114 (PMC12550195; doi:10.1016/j.resplu.2025.101114)

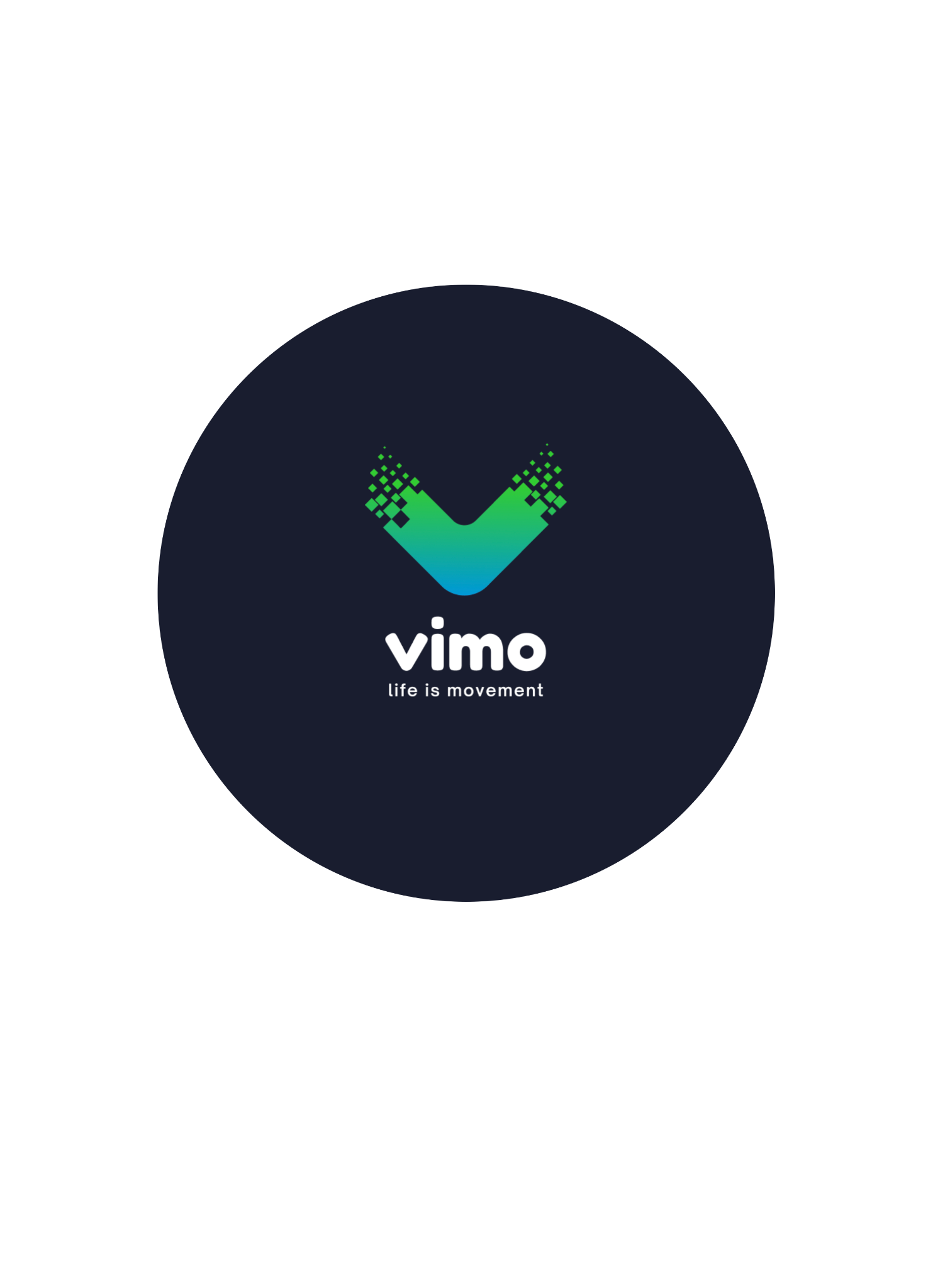


**Screens**

# Authors

Rodrigo Vieira Gardin Table of Contents

[**Authors** **2**](#_Toc3519)

[**1. Screens** **4**](#_Toc3520)

[1.1 Start Session 4](#_Toc3521)

[1.2 Session 5](#_Toc3522)

[1.3 Seings 6](#_Toc3523)

[1.4 Start Calibration 7](#_Toc3524)

[1.5 Drift Calibration 8](#_Toc3525)

[1.6 Drift Calibration Success 9](#_Toc3526)

[1.7 Drift Calibration Warning 10](#_Toc3527)

[1.8 Gain Calibration 11](#_Toc3528)

[1.9 Confirmation Screen 12](#_Toc3529)

## 1. Screens

### 1.1 Start Session


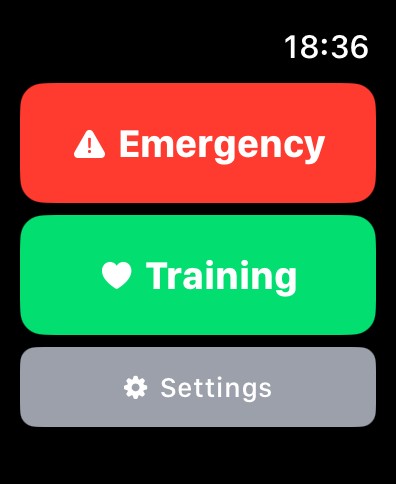


### 1.2 Session


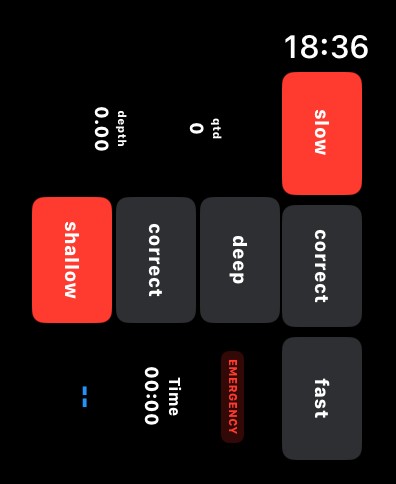


### 1.3 Seings


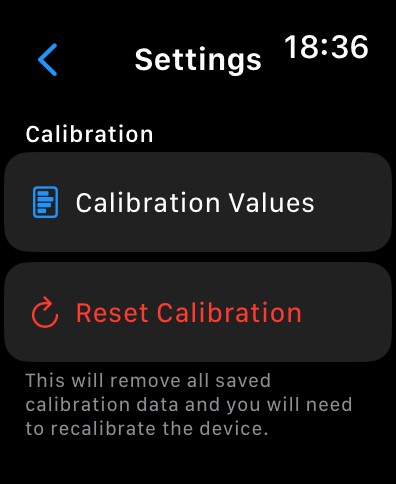


### 1.4 Start Calibration


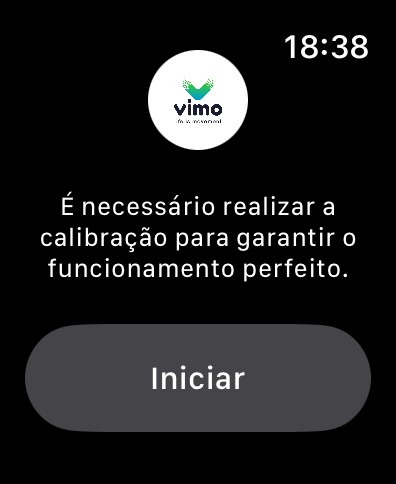


### 1.5 Drift Calibration


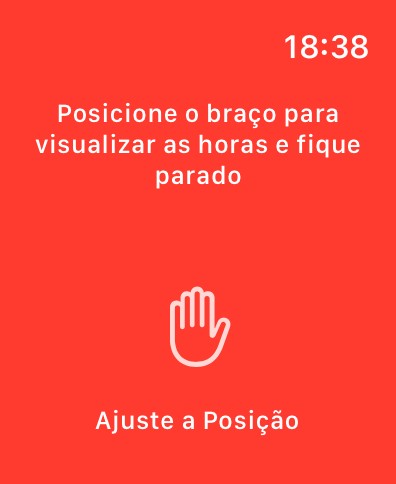


### 1.6 Drift Calibration Success


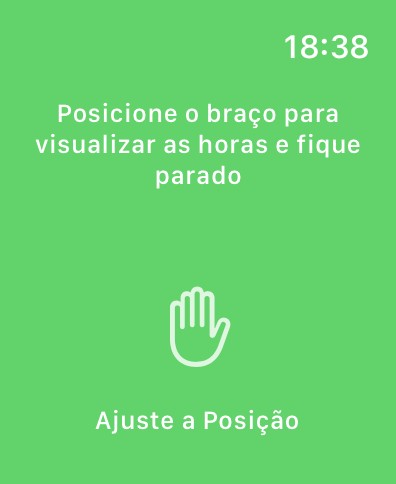


### 1.7 Drift Calibration Warning


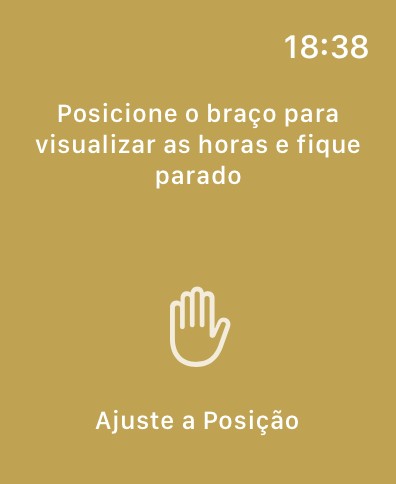


### 1.8 Gain Calibration


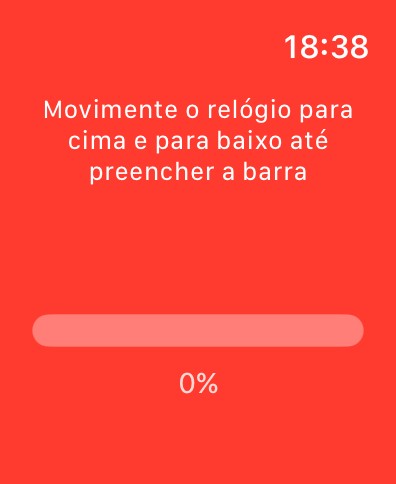


### 1.9 Confirmation Screen


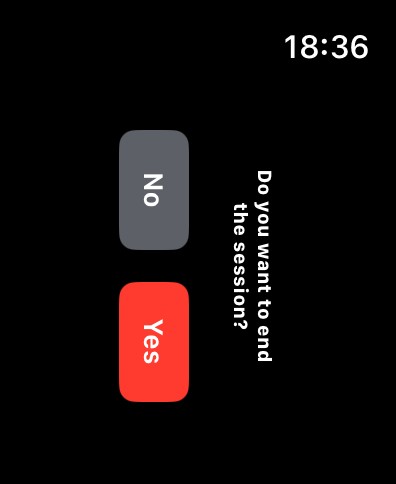

Supplement: Supplementary Data 11 [file mmc11.docx]
