# Supplementary material for: Effectiveness of a smartwatch-based feedback system in improving cardiopulmonary resuscitation quality: a simulation study
Source: Resusc Plus. 2025 Sep 30;26:101114. doi: 10.1016/j.resplu.2025.101114 (PMC12550195; doi:10.1016/j.resplu.2025.101114)
Supplement: Supplementary Data 12 [file mmc12.docx]

Supplementary Archives Titles:

Figure 6. Average compression rate with and without smartwatch feedback.

Figure 7. Total number of compressions with and without smartwatch feedback.

Table 2. Correct compression rate by CPR training level.

Table 3. Optimal displacement and average depth.

Table 4. Main outcomes.

Table 5. Main outcomes by CPR training level.

Table 6. Main outcomes by professionals.

Table 7. Main outcomes by experience level.

Table 8. Full descriptive parameters.
